# Immunotherapy in chronic lymphocytic leukemia: advances and challenges

**DOI:** 10.1186/s40164-025-00644-5

**Published:** 2025-04-10

**Authors:** Pan Gao, Yang Zhang, Jun Ma, Ya Zhang

**Affiliations:** Department of Hematology, Shandong Provincial Hospital Affiliated to Shandong First Medical University, No.324, Jingwu Road, Jinan, Shandong 250021 China

**Keywords:** Chronic lymphocytic leukemia, Immunotherapy, Antibody, Immune cell, Combination therapy

## Abstract

Chronic lymphocytic leukemia (CLL) is characterized as a clonal proliferation of mature B lymphocytes with distinct immunophenotypic traits, predominantly affecting the middle-aged and elderly population. This condition is marked by an accumulation of lymphocytes within the peripheral blood, bone marrow, spleen, and lymph nodes. The associated immune dysregulation predisposes CLL patients to a higher risk of secondary malignancies and infections, which significantly contribute to morbidity and mortality rates. The advent of immunotherapy has revolutionized the prognosis of CLL, advancing treatment modalities and offering substantial benefits to patient outcomes. This review endeavors to synthesize and scrutinize the efficacy, merits, and limitations of the current immunotherapeutic strategies for CLL. The aim is to inform the selection of optimal treatment regimens tailored to individual patient needs. Furthermore, the review juxtaposes various therapeutic combinations to elucidate the comparative advantages of each approach, with the ultimate objective of enhancing patient prognosis and quality of life.

## Introduction

Chronic Lymphocytic Leukemia (CLL) is a form of chronic leukemia distinguished by the presence of neoplastic B lymphocytes and a propensity for systemic lymphadenopathy [1, 2]. This condition is marked by the infiltration of blood and bone marrow in affected individuals, whereas Small Lymphocytic Lymphoma (SLL) is primarily identified by lymph node enlargement. Furthermore, CLL patients often exhibit immune dysregulations, including hypoalbuminemia and autoimmune phenomena, with a particular prevalence of autoimmune hemolytic anemia [3, 4].

Over the past decade, significant strides have been made in the therapeutic management of CLL [5]. The treatment paradigm has shifted from traditional chemoimmunotherapy (CIT) to more targeted and biologically based therapies [6]. The immunotherapy landscape continues to expand with innovative approaches such as Chimeric Antigen Receptor (CAR) cell therapy [7, 8], bispecific antibody ( BsAb) therapy, and Antibody-Drug Conjugates (ADCs) therapy, which are set to be detailed in this article. The evolution of CLL treatment is further underscored by the historical progression from the approval of Rituximab and Alemtuzumab [9] to the initiation of clinical trials for novel monoclonal antibodies targeting CD20 and CD23, as well as the advent of innovative immunotherapeutic strategies, indicating a profound transformation in the field [10].

**Fig. 1:**
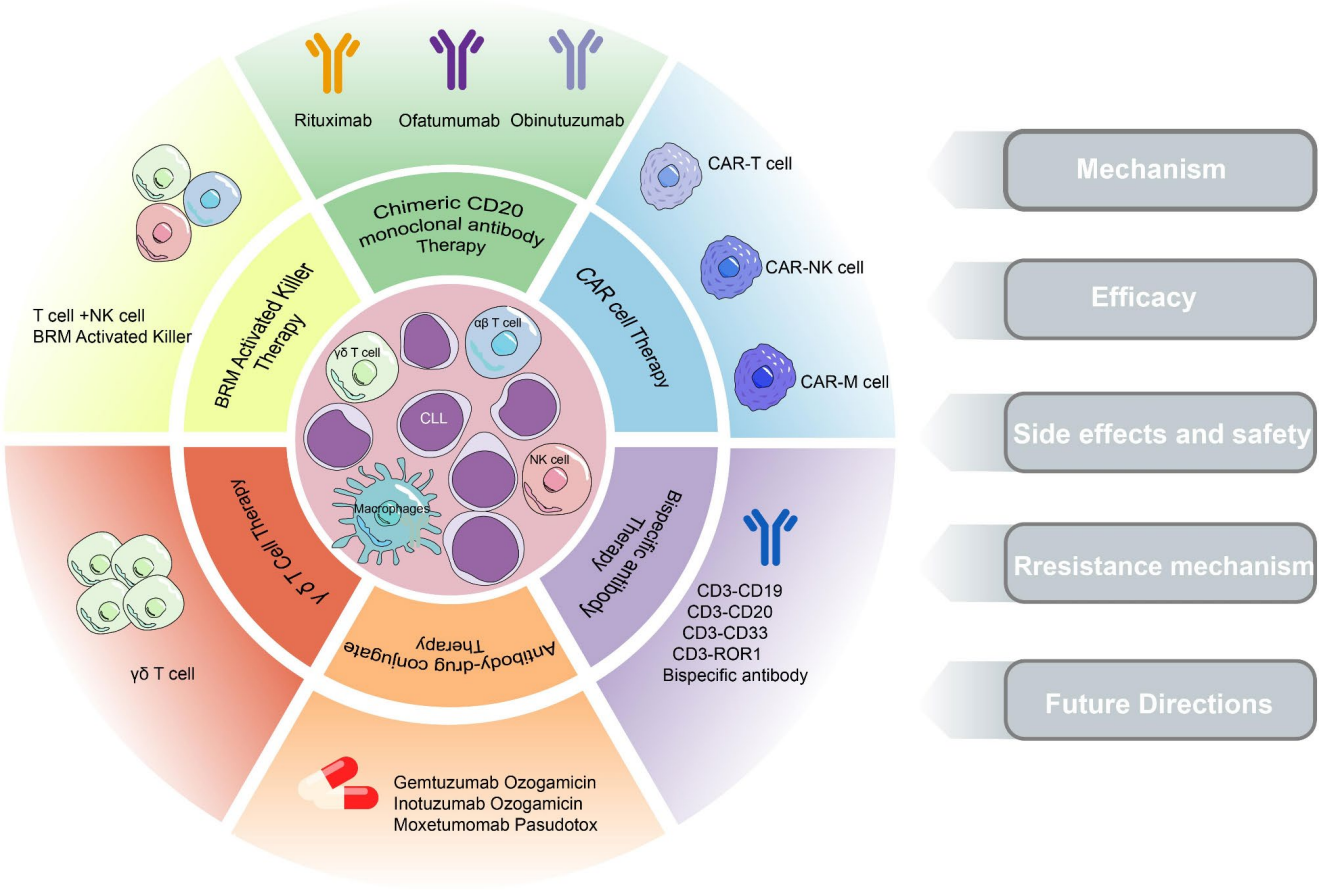
The immunotherapy of CLL mainly includes the following six aspects. They are Chimeric CD20 monoclonal antibody therapy; CAR cell therapy; Bispecific antibody therapy; Combination therapy with antibody drugs; γδ T cell therapy; and BAK cell therapy. We will discuss five aspects: mechanism, efficacy, side effects and safety, resistance mechanism, and future directions

The primary aim of this study is to provide a comprehensive overview of the most recent advancements in immunotherapy for CLL. This includes an in-depth analysis of the mechanisms of action, therapeutic efficacy, and challenges associated with CD20 monoclonal antibodies (mAbs), CAR cell therapy, bispecific antibodies (BsAbs), and other emerging therapeutic approaches. Moreover, the study investigates the potential synergistic effects of various combination immunotherapy regimens, thereby expanding the range of treatment options available for CLL immunotherapy (Fig. 1).

## Chimeric CD20 monoclonal antibody therapy

### Mechanism of CD20 mAbs

**Fig. 2:**
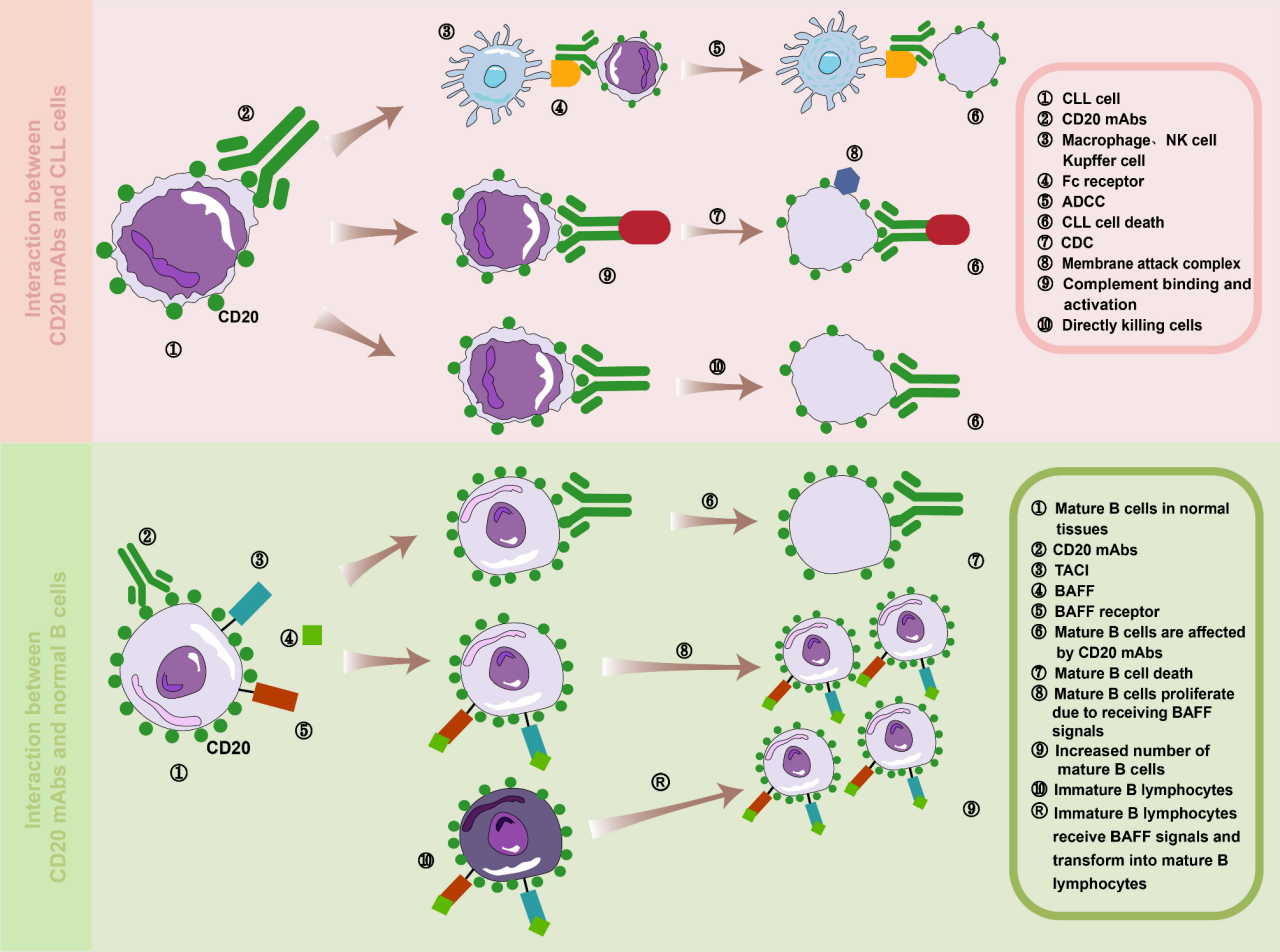
The reaction between anti-CD20 monoclonal antibody and CLL cells. CD20 mAbs mainly induce the death of tumor cells expressing CD20 molecules through three mechanisms, namely antibody-dependent cytotoxicity (ADCC), complement-dependent cytotoxicity (CDC), and direct effect killing of tumor cells caused by the binding of antibodies to CD20 molecules. In addition, this picture shows the expression of CD20 in normal B cells and CLL cells. Normal B cells typically express high-density CD20 molecules, while CLL cells have lower levels of CD20 expression. This difference may influence the therapeutic efficacy of CD20 monoclonal antibodies. BAFF-R, BAFF-Receptor

CD20 is a membrane protein specific to B-cells, which is highly expressed on the surface of B-cell-derived lymphomas, leukemias, and other neoplastic cells. Monoclonal antibodies targeting CD20 play a pivotal role in the targeted therapy of these malignant tumors and autoimmune disorders [11]. Current research indicates that CD20 mAbs primarily induce tumor cell demise through three principal mechanisms: antibody-dependent cellular cytotoxicity (ADCC) [12], complement-dependent cytotoxicity (CDC) [13], and direct effects mediated by the antibody binding to CD20 molecules. These direct effects encompass the inhibition of cellular proliferation, modulation of the cell cycle, and the induction of apoptosis [14, 15]. Furthermore, it has been observed that while CD20 mAbs can affect normal B cells due to their expression of CD20, naive normal B cells often recover and repopulate B-cell compartments following the elimination of CD20-expressing lymphoma cells by the mAbs [16](Fig. 2). Grasping the intricacies of how CD20 monoclonal antibodies interact with B cells and modulate immune responses lays the groundwork for understanding their clinical impact. We will delve into this impact in the subsequent section on clinical outcomes.

### Efficacy of CD20 mAbs

Rituximab, a murine-derived monoclonal antibody (mAb), represents the first generation of CD20-targeting mAbs used in the treatment of CLL and other maliganancies [17]. It specifically binds to the CD20 antigen and has demonstrated significant efficacy in leukemia, lymphoma, and rheumatoid arthritis [18]. Since its approval for CLL treatment in 2010, rituximab has transformed therapeutic approaches [19], increasing the complete response rate (CR) by 21% and prolonging median progression-free survival (PFS) by 5.1 months in a clinical trial involving non-Hodgkin lymphoma (NHL) [20]. In CLL, it achieved a combined CR and partial response rate (PR) of 66% and a median PFS of 10.1 months [21].

Ofatumumab, a fully humanized CD20-targeting mAb, represents the second generation [22] and is particularly effective in patients resistant to rituximab. In a clinical trial focused on refractory CLL, ofatumumab monotherapy achieved an overall CR and PR rate of 28%, and a median PFS of 5.7 months [23]. Additionally, it has shown potential in reducing relapse rates in multiple sclerosis (MS) compared to standard chemotherapy regimens [24].

Obinutuzumab, the third-generation CD20 mAb [25], is engineered to enhance ADCC through glycosylation of its fragment crystallizable (Fc) segment. This enhancement allows it to more efficiently activate FcγRIIIa (CD16a) and FcγRIa (CD64) compared to rituximab [26, 27]. In clinical trials, obinutuzumab monotherapy significantly prolonged median PFS by 13.8 months in previously untreated follicular lymphoma (FL) patients compared to chemotherapy [28], and in relapsed/refractory (R/R) CLL, it demonstrated a superior combined CR and PR rate with a median PFS of 14.2 months [29].

### CD20 mAbs combination therapy

In recent years, numerous studies have demonstrated that the combination of rituximab with other therapeutic agents often results in superior therapeutic outcomes compared to rituximab monotherapy [30]. For instance, a comparative analysis revealed that in untreated CLL patients aged 70 years or younger, the ibrutinib-rituximab regimen conferred superior PFS and overall survival (OS) compared to the standard chemoimmunotherapy regimen of fludarabine, cyclophosphamide, and rituximab (FCR) [31, 32]. Additionally, ibrutinib-rituximab was found to be superior in terms of PFS among untreated elderly CLL patients compared to the bendamustine-rituximab combination [33, 34]. Another study indicated that ibrutinib combined with obinutuzumab significantly prolonged median PFS [35]. These findings suggest that combining CD20 mAbs with Bruton’s tyrosine kinase (BTK) inhibitors, such as ibrutinib, yields a more potent therapeutic effect.

Further research compared traditional chemotherapy and immunotherapy with the bendamustine-rituximab combination, indicating that FCR remains the preferred first-line treatment for CLL patients, despite the lower toxicity of bendamustine-rituximab [36]. The fixed-duration regimen of venetoclax-rituximab (VenR) has also been shown to enhance PFS in individuals with R/R CLL [37, 38]. Recent studies revealed that ibrutinib combined with rituximab enhances anti-CLL T cell cytotoxicity, shifting from CD4 + T cell immune synapse to increased CD8 + lytic synapse during therapy [39]. Collectively, these investigations underscore the superior efficacy of rituximab when paired with other therapeutic agents compared to its use as a monotherapy(Table 1).

**Table 1:** CD20 mAbs in chronic lymphocytic leukemia(CLL) and their combined application

Drug regimen	Type	Mechanism	ORR	PFS (months)	OS (months)	Line of therapy	Main indication
Rituximab	Chimeric antibody	Modulate intracellular signaling cascades to trigger apoptosis; Regulate the expression of cyclins to suppress cell proliferation; Activate the complement system to form MAC, function as CDC; Activate immune effector cells, function as ADCC; Downregulate anti-apoptotic proteins to enhance drug sensitivity.	45%	18.6	-	First line	NHL, include FL and DLBCL; CLL, particularly R/R CLL; RA, SLE, ITP, AAV.
Ofatumumab	Fully human antibody	Modulate intracellular signaling cascades to trigger apoptosis; Activate the complement system to form MAC, function as CDC; Activate immune effector cells, function as ADCC;	51%	21	13.7	Second line	CLL, particularly in patients who have not responded to Rituximab; RMS.
Obinutuzumab	Fully human antibody	Exert direct cytotoxicity, induce apoptosis directly; After glycosylation modification, the ADCC effect was enhanced; The CDC effect is relatively modest, yet remains effective; Modulate the immune system to enhance therapeutic efficacy.	67%	14.2	-	Second line	CLL, particularly in patients who have not responded to Rituximab; R/R FL.
FCR	-	Inhibit DNA synthesis and repair, interfere with cell proliferation; Disrupt intracellular metabolic pathways, leading to apoptosis; Alkylating agents cause DNA strand breaks and structural damage; Exert immunosuppressive effects to reduce immune-mediated injury; Mechanism of action of Rituximab; Synergistic effects, enhance therapeutic efficacy, overcome drug resistance.	91.4%	56.8	-	First line	CLL, particularly in newly diagnosed patients with good physical condition.
Bendamustine + Rituximab	Alkylating agent + Chimeric antibody	Alkylating agents cause DNA strand breaks and structural damage; Exert immunosuppressive effects to reduce immune-mediated injury; Mechanism of action of Rituximab; Synergistic effects, enhance therapeutic efficacy, overcome drug resistance.	87.5%	30	6.7	Second line	FL, particularly in older patients; CLL, especially in older patients with compromised health; DLBCL, MCL.
Venetoclax + Rituximab	Bcl-2 inhibitor + Chimeric antibody	Inhibit the Bcl-2 protein, prevent its interaction with pro-apoptotic proteins; Promote the activation of pro-apoptotic proteins, triggering apoptosis; Mechanism of action of Rituximab; Synergistic interactions, enhance the anti-apoptotic effect and overcome drug resistance.	93.3%	53.6	60	Second line	CLL, particularly R/R CLL, primary CLL, SLL.
Obinutuzumab + Ibrutinib	Fully human Antibody + BTK inhibitor	Mechanism of action of Obinutuzumab; Inhibit BTK activity to block the BCR signaling pathway, thereby inhibiting cell survival and proliferation; Inhibit cell migration and proliferation to reduce tumor spread; Synergistic effects, improve treatment efficacy and reduce MRD.	92%	-	33.3	First line	CLL, especially adult patient; SLL.
Obinutuzumab + Chlorambucil‌	Fully human Antibody + Alkylating agent	Alkylating agents destroy DNA structure, thereby inhibiting cell proliferation and division; Mechanism of action of Obinutuzumab; Induce cell cycle arrest and promote apoptosis; Enhance the anti-tumor effect, improve the remission rate.	86%	26.7	-	First line	CLL, especially in elderly patients with comorbidities.
Obinutuzumab + Venetoclax	Fully human Antibody + Bcl-2 inhibitor	Mechanism of action of Obinutuzumab; Inhibit the Bcl-2 protein, prevent its interaction with pro-apoptotic proteins; Promote the activation of pro-apoptotic proteins, triggering apoptosis; Complementary effect, deep remission, limited course of treatment.	100%	34.8	56.1	First line	CLL/SLL, include newly diagnosed patients and R/R patients.
Obinutuzumab + Acalabrutinib	Fully human Antibody + BTK inhibitor	Mechanism of action of Obinutuzumab; Block the BCR signaling pathway, thereby inhibiting cell survival and proliferation; Inhibit Btk to block B cell proliferation, transport, chemotaxis, and adhesion; Complementary effect, improve treatment efficacy, limited course of treatment.	100%	-	-	First line	CLL, include newly diagnosed patients and R/R CLL.

AAV, ANCA-associatedvasculitis; ADCC, Antibody-dependent cellular cytotoxicity; ANCA, Antineutrophil cytoplasmic antibodies; BCL, B-cell lymphoma; BTK, Bruton’s tyrosine kinase; CDC, Complement-dependent cytotoxicity; CLL, Chronic lymphocytic leukemia; CR, Complete response rate; DLBCL, Diffuse large B-cell lymphoma; FCR, Fludarabine + cyclophosphamide + rituximab; FL, Follicular lymphoma; ITP, Immune thrombocytopenia; mAbs, Monoclonal antibodies; MAC, Membrane attack complex; MCL, Mantle cell lymphoma; MRD, Minimal residual disease; NHL, Non-Hodgkin lymphoma; ORR, Objective response rate; OS, Overall survival; PFS, Progression-free survival; PR, Partial response rate; RA, Rheumatoid arthritis; RMS, Relapsed multiple sclerosis; R/R, Relapsed/refractory; SLE, Systemic lupus erythematosus; SLL, Small lymphocytic lymphoma

### Factors influencing the efficacy of CD20 mAbs

The efficacy of CD20 mAbs in treatment is influenced by a multitude of factors, which can be broadly categorized into two main aspects: on the one hand, there are individual differences between patients, and on the other hand, there are also typing differences between diseases.

The difference between individual patients is the main reason for the difference in the efficacy of CD20 mAbs. The individual differences between patients mainly come from the following aspects: The first is the difference caused by genetic factors, some studies have shown that some heritable genes will also lead to different effects of CD20 mAbs. For example, differences in the FC-γ receptor gene are associated with the efficacy of rituximab [40]. The second is the patient’s immune status. Because CD20 mAbs rely on the immune system to function, if the patient’s immune response is low, the efficacy of CD20 mAbs will naturally be affected [41]. Ultimately, the possible factor affecting the efficacy of CD20 mAbs is its pharmacokinetics. For example, enzymes related to drug metabolism, if they are produced too much and degraded too slowly, will adversely affect the efficacy of CD20 mAbs [42].

Another reason is that different subtypes of the disease have different effects on the efficacy of CD20 mAbs. Several distinct molecular subtypes of CLL can influence the efficacy of CD20 mAbs. For instance, certain CLL subtypes may harbor distinct genetic variations, such as TP53 gene mutations or chromosomal aberrations (e.g., deletions of 11q and 17p), which could potentially modulate the expression levels of CD20 molecules or impact cellular signaling pathways. These genetic disparities may, in turn, affect the binding and functionality of CD20 mAbs [43].

For patients with CLL, the differential expression levels of CD20 across various CLL subtypes may be a primary influencing factor. One of the main mechanisms of action of CD20 mAbs is their binding to CD20 molecules. If a particular CLL subtype exhibits low CD20 expression, the efficacy of these antibodies may be compromised. For instance, CLL cells with specific genetic variations may express lower levels of CD20, thereby reducing the effectiveness of CD20 mAbs [44]. Following that, CLL subtypes may feature distinct signaling pathways. Given that the efficacy of CD20 mAbs may depend on specific signaling pathways, these differences can influence therapeutic outcomes. For instance, the activation or inhibition of certain signaling pathways may modulate the cytotoxic response elicited by CD20 mAbs [45]. A final factor regarding the different subtypes of CLL is the Tumor microenvironment (TME), which includes tumor-associated T cells, macrophages, and other cells, and the presence of these cells may also have a certain impact on the efficacy of CD20 mAbs [46].

In the treatment of CLL, the intricate nature of the TME exerts a substantial influence on therapeutic outcomes. The depletion of immune cells and the secretion of inhibitory cytokines within the TME are pivotal factors influencing treatment response. For instance, T cell exhaustion is more prevalent among CLL patients, characterized by a progressive decline in T cell functionality. This phenomenon may be associated with the overexpression of inhibitory receptors such as PD-1 [47]. Moreover, inhibitory cytokines within the TME, such as TGF-β and IL-10, may further diminish the immune response and facilitate tumor immune evasion [48]. In addition, other components within the TME, such as tumor-associated macrophages (TAMs) and myeloid-derived suppressor cells (MDSCs), may also contribute to tumor progression by secreting inhibitory cytokines or directly suppressing T cell activity. These mechanisms collectively render the treatment of CLL highly complex and challenging [49]. To surmount these challenges, future research might focus on developing therapeutic strategies targeting the TME. For instance, restoring T cell function via immune checkpoint inhibitors or enhancing treatment efficacy by modulating the immune microenvironment of the TME could be promising approaches [50]. Strategies targeting inhibitory cytokines, such as TGF-β, have demonstrated the potential to bolster anti-tumor immunity [51]. Moreover, combination therapy involving immune checkpoint inhibitors has also shown promise in preclinical and clinical research [52].

### Side effects and safety of CD20 mAbs

CD20 mAbs have become a cornerstone in the first-line treatment of NHL, CLL, and certain autoimmune diseases, demonstrating significant efficacy against hematologic neoplasms [53]. Despite these therapeutic successes, the use of CD20 mAbs is not without adverse effects. Consequently, it is imperative for clinicians to be cognizant of these potential complications and to implement preemptive safety measures to mitigate the risk of adverse events.

The administration of CD20 mAbs can be accompanied by several adverse effects, which are primarily categorized as follows: The first is acute anaphylaxis, which is characterized by fever, chills, nausea, vomiting, rash, and other symptoms. In extreme cases, anaphylactic shock may ensue, necessitating immediate clinical intervention [44]. The incidence rate of acute anaphylaxis associated with CD20 mAb therapy is approximately from 5–10% [54]. CD20 mAbs can trigger the release of cytokines, leading to cytokine release syndrome (CRS). This syndrome typically manifests with fever, hypotension, respiratory distress, and altered mental status, and in severe instances, it can be life-threatening [55]. The incidence rate of CRS in patients receiving CD20 mAbs ranges from 10–20% [56]. Some patients also develop serum disease, which is caused by the immune system’s inflammatory response to foreign antibodies, and patients mainly present with rashes and swollen lymph nodes. The incidence of serum sickness-like reactions is around from 5–15% [57]. In more severe cases, rapid cell lysis induced by CD20 mAbs can result in tumor lysis syndrome (TLS), a condition that may lead to complications such as hyperkalemia and hyperuricemia [58]. The incidence of TLS is relatively low, estimated at from 1–5% [59].

In addition to the acute adverse effects, long - term use of CD20 mAbs may also lead to chronic complications. These include prolonged immunosuppression, which can increase the risk of infections. The incidence of serious infections in patients receiving long - term CD20 mAb therapy is approximately from 10–15% [60]. Other long - term adverse effects may include autoimmune - like reactions, such as thrombocytopenia and hemolytic anemia, with an incidence rate of around from 2–5% [61]. There is also a potential risk of secondary malignancies, although the incidence is relatively low and varies depending on the duration of treatment and other risk factors [62].

Given the potential side effects associated with CD20 mAb therapy, clinicians must exercise caution and implement a series of preemptive safety measures. Firstly, it is essential to conduct timely monitoring of patient’s vital signs following drug administration to facilitate prompt response to any adverse reactions. Additionally, administering prophylactic medications in a targeted manner, such as anti-allergy drugs, can help mitigate the risk of anaphylactic reactions [28]. For patients who experience side effects, it may be necessary to reduce the dosage or discontinue the treatment immediately [29]. In cases of patients with highly aggressive tumors that are at risk of TLS, preemptive measures such as urine alkalinization or hydration should be administered to prevent the onset of TLS [35]. Furthermore, since patients undergoing CD20 mAb therapy are more immunocompromised than the general population, it is crucial to take infection prevention measures throughout the treatment course, including the use of prophylactic antibiotics and vaccinations where appropriate [42]. Ultimately, clinical evidence suggests that certain antibodies possess cardiotoxic potential. Consequently, it is imperative to monitor cardiac function in patients, particularly those with pre-existing weakness or cardiac dysfunction, following the initiation of treatment [63].

In conclusion, CD20 mAbs have demonstrated remarkable efficacy and have established an indispensable role in the therapeutic management of CLL, both as monotherapy and in combination with other treatments. Nonetheless, the performance of CD20 mAbs is influenced by a multitude of factors, and their clinical application is not without certain side effects. Consequently, there is a continued need for research aimed at identifying and addressing the factors that can stabilize the efficacy of CD20 mAbs. Future efforts should be directed towards enhancing the therapeutic effectiveness and safety profiles of these mAbs, ensuring that they continue to serve as a cornerstone in the treatment armamentarium for CLL.

## Chimeric antigen receptor cell therapy

### Mechanism of CRA-T cell

As therapeutic strategies for CLL continue to advance, traditional chemotherapy alone is increasingly insufficient to address the complexities of CLL treatment. In this context, innovative cell therapies are demonstrating significant progress [64, 65]. CAR cell therapy, a subtype of adoptive immunotherapy [66], is defined as a therapeutic approach involving the adoptive transfer of cells with anti-tumor activity to a tumor-bearing host, with the aim of modulating and promoting tumor regression.

CAR-T cell therapy, a form of passive immunotherapy, represents a novel and highly specific targeted approach to tumor cell destruction [67]. The therapeutic principle involves the ex vivo collection of T cells from a patient, followed by their genetic modification to express a CAR. This engineered receptor enables the T cells to recognize and bind to specific antigens present on the surface of tumor cells, thereby directing the T cells to accurately identify and eliminate these malignant cells. The genetically modified CAR-T cells are then expanded and reinfused into the patient to initiate anti-tumor activity. In the context of CLL, cells often display B cell surface markers such as CD19, CD20, CD22, and CD23, which serve as potential targets for CAR-T cell therapy [68, 69]. Among these, CD19 is the most frequently utilized target due to its high expression levels and relative specificity on CLL cell surfaces [70]. Upon binding to CLL cell surface antigens, the transmembrane signal transduction domain of the CAR engages intracellular signaling pathways, leading to T cell activation. Activated CAR-T cells secrete cytokines, including interferon-γ (IFN-γ) and tumor necrosis factor-α (TNF-α), which contribute to the proliferation and further activation of CAR-T cells [71].

**Fig. 3:**
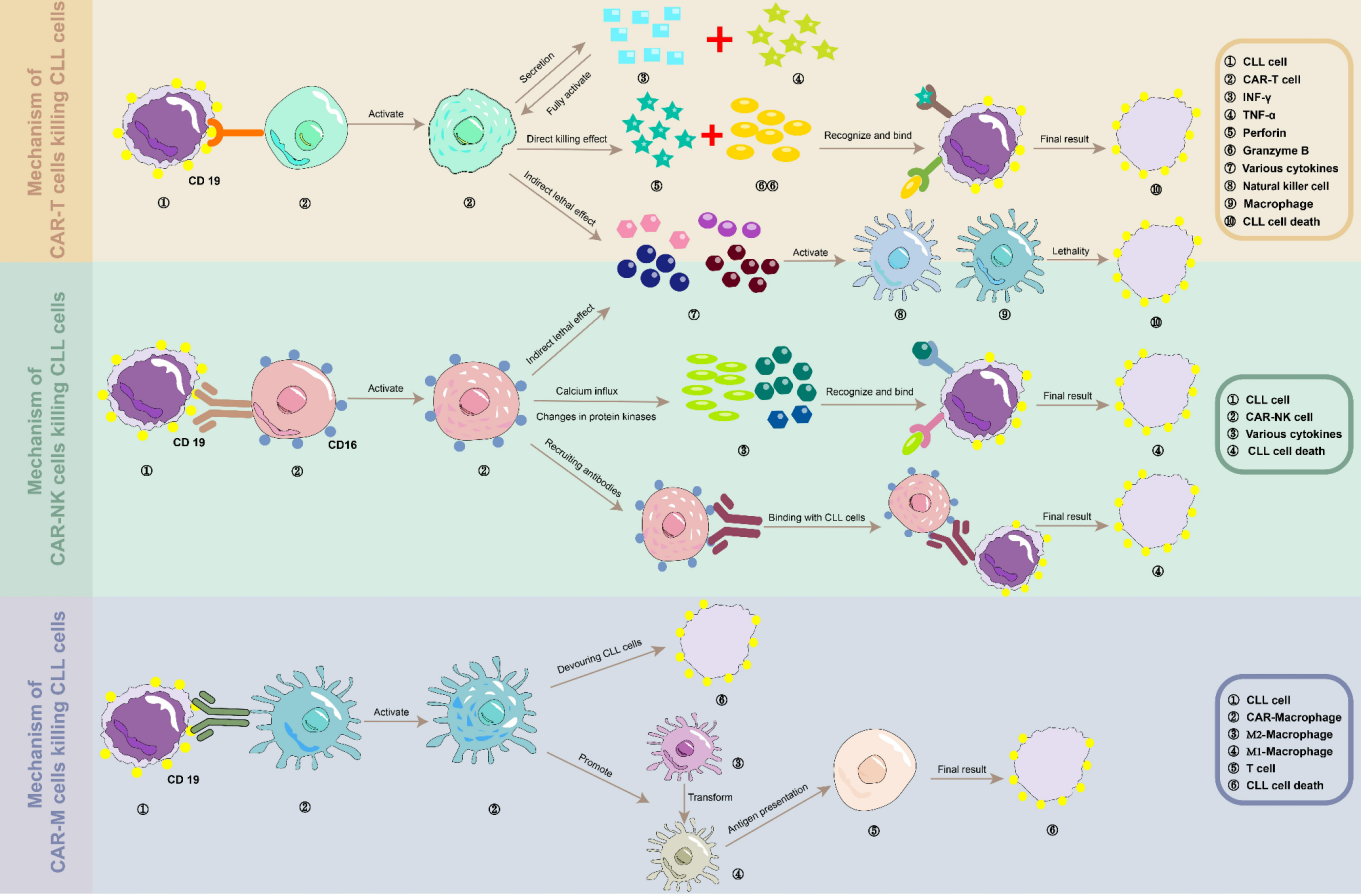
**The reaction between CAR cells and CLL cells.** Mainly including CAR-T cells, CAR-NK cells, and CAR-M cells

The cytotoxic effects of CAR-T cells on CLL cells are primarily mediated through two distinct mechanisms: direct and indirect killing. Direct killing is primarily executed through the release of perforin and granzyme B from CAR-T cells, which induce apoptosis in CLL cells. Indirect killing, on the other hand, involves the activation of endogenous immune cells by cytokines secreted by CAR-T cells **(**Table 2**)**. This activation enhances the engagement of natural killer (NK) cells and macrophages in the collective immune response against CLL cells [70](Fig. 3). Understanding these mechanisms is crucial for appreciating the therapeutic potential of CAR-T cells, which we will explore further in the following section on clinical outcomes.

**Table 2:** Application of CAR-T cell therapy in CLL

Mechanism category	Specific mechanism	Function description	Target Antigen	Response Rate (ORR/CR)
Direct toxic effects	Perforin and granzyme mediated	After CAR-T cells recognize and bind to target cells, they release perforin to “drill holes” on the surface of tumor cells, and granzyme enters the interior of tumor cells, inducing their apoptosis.	CD19, CD20, CD22, CD79a, CD23	ORR: 65%, CR: 25%
	Fas-FasL mediated	CAR-T cells induce tumor cell apoptosis through the Fas-FasL axis.	CD19, CD20, CD22	ORR: 60%, CR: 20%
Cytokine secretion	Enhance anti-tumor activity	Activated CAR-T cells secrete cytokines such as TNF-α, IFN-γ, IL−2, IL−6, enhancing their anti-tumor activity.	CD19, CD20, CD22	ORR: 70%, CR: 30%
	Recruit other immune cells	The secreted cytokines can activate and recruit NK cells, B cells, macrophages, etc. to participate in anti-tumor reactions.	CD19, CD20, CD22	ORR: 75%, CR: 35%
Indirect anti-tumor effect	Activate macrophages	CD4 + CAR-T cells secrete IFN-γ to activate macrophages and enhance their phagocytic function.	CD19, CD20, CD22	ORR: 65%, CR: 25%
	Upregulation of MHC I molecules	IFN-γ can upregulate the expression of MHC class I molecules on the surface of tumor cells, enhancing their sensitivity to the immune system.	CD19, CD20, CD22	ORR: 60%, CR: 20%
Overcome immune suppression	Secreting immune stimulating cytokines	Armored CAR-T cells can secrete IL−12 or IL−18, reshape the tumor microenvironment, and resist immune suppression.	CD19, CD20, CD22	ORR: 70%, CR: 30%
	Joint immune checkpoint inhibitors	CAR-T cells combined with anti-PD−1/PD-L1 antibodies overcome tumor cell mediated immune suppression through PD-L1.	CD19, CD20, CD22	ORR: 75%, CR: 35%
Enhance cell viability	Pre treatment chemotherapy	Administering chemotherapy drugs such as cyclophosphamide and fludarabine prior to CAR-T cell infusion depletes regulatory T cells and myeloid-derived suppressor cells, thereby increasing CAR-T cell activity.	CD19, CD20, CD22	ORR: 80%, CR: 40%
	Combination drug therapy	Combined with drugs such as ibrutinib, this approach promotes CAR-T cell proliferation and metabolism, reduces inflammatory reactions, and enhances therapeutic efficacy.	CD19, CD20, CD22	ORR: 70%, CR: 30%

CAR, Chimeric antigen receptor; CR, Complete response; FASL, Fas ligand; IFN-γ, Interferon-γ; IL-2, Interleukin−2; IL-6, Interleukin−6; IL-12, Interleukin−12; IL-18, Interleukin−18; ORR, Objective response rate; PD-1, Programmed Death−1; PD-L1, Programmed Death-Ligand 1; TNF-α, Tumor necrosis factor-α

### Efficacy of CAR-T cell

Building on the mechanistic insights discussed above, we now turn to the clinical outcomes of CAR-T cell therapy, which highlight its real-world impact and therapeutic potential. CAR-T cell therapy has revolutionized the treatment paradigm for hematological malignancies, demonstrating the ability to induce durable CR and achieving high ORR in patients with refractory disease who have undergone multiple lines of therapy. For instance, a multicenter study of CD19-targeted CAR-T cell therapy in patients with Richter’s transformation (RT) from CLL demonstrated a median OS of 25.8 months for the entire cohort, with a notably shorter median OS of 12.3 months for patients who had received prior non-CIT CLL treatment. This study included 309 patients with RT, of whom 55 received CAR T-cell therapy for RT. The response was captured for 51 (92.7%) patients, with an ORR of 62.7% (complete response [CR] *n* = 23, partial response [PR] *n* = 9, progressive disease [PD]/Death *n* = 19), and after a median follow-up of 15.3 months, the median OS was 9.0 months (95% CI: 6.6–20.7) from CAR T-cell therapy [72].

Additionally, a study focusing on approved CD19-targeted CAR-T cell therapies in patients with R/R CLL reported an ORR of 70% and a CR rate of 30%, with a median PFS of 12 months. This study was a retrospective analysis of patients treated with CD19-targeted CAR-T cells, and the specific number of patients and other detailed information were not mentioned [73]. These findings highlight the potential of CAR-T cell therapy in improving outcomes for patients with CLL and its aggressive complications [74].

### CAR-T cell combination therapy

Having explored the significant impact of CAR-T cell therapy as a standalone treatment modality, we now turn our attention to the emerging strategies aimed at enhancing its efficacy, addressing the challenges encountered in clinical practice, and maximizing patient outcomes. Recent clinical studies have revealed that the absence of CTLA4 can restore T-cell functionality in leukemia patients who have previously not responded to CAR-T cell therapy. For example, a single-arm, open-label clinical trial involving 20 patients with R/R CLL demonstrated that CTLA4 depletion significantly enhanced CAR-T cell activity, achieving an ORR of 70% and a CR rate of 30% with a median PFS of 12 months. The patient population had a median age of 62 years, and all had received at least one prior treatment regimen [75]. Consequently, the targeted elimination of CTLA4 reactivates T cells in patients with CLL, offering a potential strategy to enhance their response to CAR-T cell treatments [75]. Additionally, research has demonstrated that inhibiting PI3K δ/γ can induce epigenetic and metabolic reprogramming in human CAR-T cells, thereby enhancing their anti-tumor cytotoxicity [76]. A preclinical study using a CLL mouse model showed that CAR-T cells with suppressed PI3Kδ/γ significantly improved survival rates, with a median OS extended to 18 months compared to conventionally manufactured CAR-T cells. These findings suggest that incorporating PI3K δ/γ inhibitors during the production of CD8 + CAR-T cells can enhance their efficacy in eliminating CLL in vivo [77].

Building on the advancements in enhancing CAR-T cell efficacy through targeted strategies, we now delve into the realm of combination therapies, where CAR-T cells are leveraged in conjunction with other therapeutic modalities to further amplify their therapeutic potential and address the complex challenges of cancer treatment. The combination of CAR-T cells with immune checkpoint inhibitors (ICIs) has emerged as a strategy to augment clinical benefits. Evidence suggests that ICIs can enhance the functionality of tumor-infiltrating lymphocytes and reinvigorate their capacity to target cancer cells. Consequently, when CAR-T cells are employed in conjunction with ICIs, the latter can potentiate the efficacy of the CAR-T cells within the TME, thereby potentially offering greater benefits to patients [78]. For instance, a single-arm, open-label phase II clinical trial involving patients with R/R solid tumors demonstrated an ORR of 45% and a median PFS of 6.5 months when ICIs were combined with CAR-T cells. Furthermore, CAR-T cells can be combined with CD20 mAbs and BsAbs targeting CD3/CD20. A multicenter, randomized phase II clinical trial demonstrated that the combination of anti-CD19 CAR-T cells with rituximab in patients with refractory large B-cell lymphoma (LBCL) achieved a CR rate of 65% and an overall response rate of 88%, marking a significant advancement in LBCL treatment [79]. The patient population included 120 individuals with a median age of 60 years, 60% of whom were male and had received at least two prior lines of therapy. The synergy between anti-CD19 CAR-T cells and ibrutinib has also been shown to yield higher survival and remission rates in the treatment of CLL compared to CAR-T cells alone [80, 81]. In a single-arm, open-label phase II clinical trial involving 80 patients with R/R CLL, the combination therapy achieved a CR rate of 30% and an ORR of 70%, with a median PFS of 14.2 months. The patient population had a median age of 65 years, with 65% being male and having received at least one prior treatment regimen [82]. Lastly, beyond these combinations, the exploration of other combination therapies holds broad promise [83], indicating that the development of CAR-T cell therapy in combination with other treatment modalities is a promising avenue for future research and clinical application.

### Adverse reactions and treatment of CAR-T cell therapy

CRS is a common and often severe adverse effect of CAR-T cell therapy, with reported incidence rates ranging from 30 to 70% depending on the specific CAR-T product and patient population. The clinical manifestations of CRS include fever, chills, hypotension, tachycardia, and in severe cases, can escalate to multi-organ dysfunction and even mortality [84]. Management strategies for CRS primarily involve pharmacological interventions and supportive care. IL-6 receptor antagonists, such as Tocilizumab, and corticosteroids are commonly used to mitigate symptoms and complications associated with CRS [85].

Immune Effector Cell-Associated Neurotoxicity Syndrome (ICANS) is another significant adverse event associated with CAR-T cell therapy, with incidence rates ranging from 10 to 30%. Clinical manifestations of ICANS includ headaches, cognitive impairment, speech disorders, and epileptic seizures [86]. Management of ICANS is largely symptomatic and supportive, involving the administration of anticonvulsants and corticosteroids to alleviate neurological symptoms [85]. In addition to the aforementioned side effects, CAR-T cell therapy can also lead to TLS, a condition characterized by the rapid breakdown of tumor cells, resulting in the release of large amounts of intracellular contents into the bloodstream. This can cause significant metabolic disturbances, including hyperkalemia, hyperphosphatemia, and hyperuricemia, which may lead to acute kidney injury and other severe complications. TLS is a critical consideration in the management of patients undergoing CAR-T therapy, requiring close monitoring and prompt intervention to mitigate its impact [87].

In addition to CRS and ICANS, CAR-T cell therapy can also lead to other adverse effects and long-term toxicities. Hematologic toxicities, such as neutropenia, thrombocytopenia, and anemia, are frequently observed following CAR-T cell infusion [88]. These cytopenias are hypothesized to result from the direct inhibitory effects of CAR-T cells on bone marrow function [89]. Furthermore, CAR-T cell therapy can cause immune suppression, increasing the risk of infections [90]. tandard management of infections includes the use of antibiotics, antivirals, or antifungals, depending on the type of infection [91].

Other immune-related adverse events (irAEs) that may occur include skin toxicities (e.g., rash, pruritus) [92], endocrine toxicities (e.g., thyroid dysfunction) [93], hepatic toxicities [94] (e.g., elevated liver enzymes), pulmonary toxicities [95] (e.g., interstitial lung disease), and cardiac toxicities [96] (e.g., myocarditis). The incidence rates of these irAEs vary but generally range from 5 to 20%, depending on the specific organ system affected. Management strategies for these irAEs typically involve corticosteroids or other immunosuppressive agents, as well as supportive care to address specific organ dysfunction [97].

### Mechanism of drug resistance and enhancement strategies of CAR-T cell for CLL

CAR-T cell therapy has demonstrated substantial efficacy in treating CLL. However, the emergence of drug resistance remains a significant challenge. Understanding the mechanisms underlying resistance is essential for developing effective strategies to overcome these obstacles. The primary mechanisms of resistance encompass the following aspects.

Firstly, tumor cells may lose the CD19 antigen through genetic or epigenetic mechanisms, rendering CAR-T cells ineffective. Research has shown that CD19 deficiency can occur through the deletion or mutation of the CD19 gene. For example, Orlando et al. reported that homozygous or biallelic frameshift mutations in CD19 are the main source of CD19 deletion and acquired CD19 resistance [98]. In addition to antigen loss, mutations in the CD19 gene can alter its structure and reduce the binding affinity of CAR-T cells. These mutations typically occur in the extracellular domain, preventing CAR-T recognition. For example, frameshift mutations in exons 2 and 4 have been found in some recurrent leukemia cases, leading to loss of CD19 protein expression [99]. Alternative splicing of the CD19 gene can produce truncated or modified forms of proteins that may not be recognized by CAR-T cells. This mechanism has been observed in several recurrent CLL cases. Significant splicing changes in recurrent leukemia include increased skipping of exons 2 and 5–6, which can result in the production of nonfunctional or altered CD19 protein isoforms that evade CAR-T cell recognition [100]. Epigenetic modifications, such as DNA methylation or histone deacetylation, can silence the expression of the CD19 gene. This silencing can be reversible, making it a potential target for epigenetic therapies [101]. CLL cells may undergo lineage switching, adopting a non-B cell phenotype that escapes CAR-T cell recognition. This mechanism highlights the need for more versatile targeting strategies [102].

To address these resistance mechanisms, several enhancement strategies have been proposed and are currently being studied.

Bispecific CAR-T cells have been developed to simultaneously target two antigens, thereby reducing the possibility of drug resistance caused by antigen loss. For example, bispecific CAR-T cells targeting both CD19 and CD20 have shown promising results in preclinical studies, demonstrating enhanced efficacy and reduced likelihood of immune escape [103]. Dual CARs involve engineering T cells to express two different CARs, each targeting a distinct antigen. This approach ensures that even if one antigen is lost, the other CAR can still mediate antitumor activity [104]. T cells redirected for universal cytokine killing (TRUCKs) are a novel type of CAR-T cell that have been engineered to secrete cytokines or immune-modulatory molecules. This enhancement allows them to recruit and activate other immune cells more effectively, thereby creating a more favorable tumor microenvironment and improving overall antitumor efficacy [105]. Identifying alternative targets, such as CD20, CD22, and CD79a, is another approach to overcoming resistance. These targets offer additional options for CAR-T cell therapy, especially in cases where CD19 is lost or mutated [106].

In conclusion, CAR-T cell-based immunotherapy has emerged as a pivotal approach in the treatment of various hematological malignancies, including ALL, Multiple Myeloma (MM), CLL, Acute Myeloid Leukemia (AML), Hodgkin’s Lymphoma (HL), and NHL. Furthermore, the field of CAR-T cell therapy for CLL patients encompasses a variety of innovative approaches, including autologous and allogeneic CAR-T cells, dual-targeting strategies, sequential infusion of dual-target CAR-T cells, and the FAST CAR-T technique. These methodologies hold promise for offering novel therapeutic options and potentially curative treatments for patients with CLL [107]. Nonetheless, the future advancement of CAR-T cell therapy is confronted with several challenges, such as understanding and overcoming drug resistance mechanisms and managing adverse reactions. Therefore, there is a critical need to proactively develop innovative CAR-T cell constructs to address these issues and further enhance the therapeutic potential of this modality.

### Mechanism of CAR-NK cell

Amidst the resounding success of CAR-T cells, CAR-NK cell therapy, which involves the genetic modification of NK cells, has emerged as a significant area of interest [108–110]. NK cells possess dual capabilities in cytotoxicity and immune regulation [111]. They exhibit potent anti-tumor activity and, unlike T cells, do not have Major Histocompatibility Complex (MHC) restrictions. They exhibit potent anti-tumor activity and, unlike T cells, do not have Major Histocompatibility Complex (MHC) restrictions. CAR-NK adoptive cell therapy (ACT) involves the transduction of NK cells with CAR genes, thereby equipping NK cells with the precision to target and recognize tumor cells [112]. These engineered cells are expanded ex vivo and subsequently administered to patients to elicit an anti-tumor response. Research indicates that CAR-NK cells can exert substantial cytotoxic effects; however, the manufacturing process of these cells is complex and requires further optimization [113].

Upon engagement with CLL cells, CAR-NK cells initiate a series of intracellular signaling cascades that activate the NK cell’s cytotoxic response. The initial step involves the binding of CAR-NK cells to CLL cells, which triggers an influx of intracellular calcium ions and alterations in protein kinase activity, thereby initiating signal transduction pathways [114]. This leads to the production of cytokines and the execution of cytotoxic effects. Subsequently, CAR-NK cells can release cytotoxic granules containing perforin and granzymes, directly inducing apoptosis in CLL cells [115]. In addition to these mechanisms, CAR-NK cells can engage with monoclonal antibodies through Fc receptors, such as CD16, which enhances their cytotoxic effect on CLL cells by facilitating ADCC [116](Fig. 3). Understanding the unique mechanisms by which CAR-NK cells target and eliminate cancer cells provides a critical foundation for appreciating their therapeutic potential. In the following section, we explore how these mechanisms translate into clinical outcomes.

### Advantages of CAR-NK cell compared with CAR-T cell

CAR-NK cells have demonstrated several advantages over CAR-T cells, which, while not diminishing the dominance of CAR-T cells in current CAR-based therapies, are increasingly establishing the significance of CAR-NK cells [117, 118]. Firstly, CAR-NK cells exhibit a lower risk of toxicity. Unlike CAR-T cells, they do not induce graft-versus-host disease (GvHD), and the incidence and severity of CRS are comparatively reduced [110]. Next, CAR-NK cells offer a higher potential for allogeneic therapy. This is attributed to the fact that NK cells do not express T cell receptors (TCRs) and are not restricted by MHC, allowing for the use of CAR-NK cells in allogeneic settings. Additionally, CAR-NK cells possess an innate killing capability that is enhanced by the specificity of the CAR. This combination results in a potent anti-tumor effect [119]. In the end, CAR-NK cells are capable of forming immune memory, enabling them to persist in the body for extended periods and combat tumor recurrence [120].

### Challenges faced by CAR-NK cells

CAR-NK cell therapy holds significant promise for cancer treatment, yet it faces substantial challenges in manufacturing and clinical application. The scalability and consistency of producing clinically effective CAR-NK cells remain critical issues, as these cells do not proliferate as extensively as CAR-T cells, potentially limiting their long-term efficacy [121]. Additionally, the immunosuppressive TME can neutralize the efficacy of CAR-NK cells, further complicating their application in solid tumors. Addressing these challenges necessitates close collaboration with regulatory agencies to ensure the safety and efficacy of CAR-NK therapies [109].

Innovative pricing and reimbursement strategies are also crucial to alleviate the economic burden on patients. Given the high costs associated with CAR-NK cell therapy, developing accessible pricing models and securing adequate reimbursement from healthcare systems can significantly improve patient access [122]. For instance, value-based pricing models that consider the long-term benefits and cost savings associated with successful treatment outcomes could be explored [123].

Looking to the future, CAR-NK cell therapy is poised for significant advancements. Ongoing research is focused on enhancing the persistence and efficacy of CAR-NK cells, identifying optimal target antigens, and integrating CAR-NK therapy with other treatments to improve outcomes. Additionally, the development of “off-the-shelf” CAR-NK cell products derived from sources such as umbilical cord blood or induced pluripotent stem cells (iPSCs) offers the potential for scalable production and reduced costs [124]. These advancements, combined with regulatory support and innovative financial strategies, will be key to realizing the full potential of CAR-NK cell therapy in cancer treatment [125].

### Mechanism of CAR-Ms

The remarkable success of CAR-T cell therapy in malignant hematological diseases, coupled with the promising potential of CAR-NK cells, has piqued research interest in the development of CAR-macrophages (CAR-Ms) for tumor immunotherapy [109]. While CAR-T cell therapy has demonstrated promising results in hematological malignancies, its efficacy in solid tumors remains a significant challenge [126–128]. Leveraging the unique effector functions of macrophages and their innate ability to infiltrate tumor tissues, researchers have genetically engineered CAR-Ms. These engineered cells have exhibited antigen-specific phagocytosis and tumor clearance capabilities in vitro studies [127] (Fig. 3).

In vitro experimental characterizations have revealed the multifaceted activities of CAR-Ms. These engineered cells have been shown to express pro-inflammatory cytokines and chemokines, facilitate the phenotypic conversion of bystander M2 macrophages to the M1 phenotype, upregulate antigen presentation mechanisms, and recruit T cells by presenting antigens to them. Additionally, CAR-Ms have demonstrated resistance to the suppressive effects of immunosuppressive cytokines. In humanized mouse models, CAR-Ms have been further observed to induce a pro-inflammatory TME and to enhance the activity of anti-tumor T cells [129, 130].

### Advantages of CAR-Ms compared with CAR-T cell

Compared to CAR-T cells, CAR-Ms possess three notable advantages. Firstly, while T cells are impeded by the physical barriers established by the tumor cell-matrix from infiltrating the TME, macrophages can effectively penetrate this environment. Tumor-associated macrophages (TAMs) are pivotal in tumor invasion, metastasis, immune suppression, and angiogenesis [128]. CAR-Ms can reduce the proportion of TAMs, modulate their cellular phenotype, and thus positively influence tumor treatment. CAR-Ms exhibit the dual capability of phagocytosing tumor cells and enhancing antigen presentation, which in turn boosts T-cell cytotoxicity [131]. Additionally, when contrasted with CAR-T cells, CAR-Ms exhibit a shorter circulation time and a reduced risk of non-tumor-targeted toxicity [132].

Despite the significant potential demonstrated by CAR-M therapy in cancer treatment, several challenges must be addressed to facilitate its clinical application. Future research endeavors should focus on the continued optimization of CAR-M design to augment their anti-tumor efficacy and minimize potential side effects. Additionally, the development of more efficient gene delivery systems and the exploration of combination therapy strategies are crucial for advancing the broad application of CAR-Ms therapy in clinical practice.

## Bispecific antibody therapy

### Mechanism of BsAbs

The therapeutic landscape for CLL has undergone a profound transformation in the last two decades, largely attributable to the advent of novel immunotherapeutic agents, such as CD20 mAbs [19]. The ongoing evolution of immunotherapy has subsequently introduced CAR therapies, which have had a significant impact on CLL treatment strategies [64, 65]. Furthermore, the emergence of BsAbs has demonstrated remarkable efficacy and is currently regarded as one of the most promising immunotherapeutic approaches for lymphoma [7, 133, 134].

BsAbs, also referred to as bifunctional antibodies, possess the unique capability to recognize and bind simultaneously to two distinct antigens or epitopes, thereby blocking multiple signaling pathways and inhibiting the growth and survival of CLL cells [135, 136]. Moreover, BsAbs can engage one arm with specific antigens on the surface of CLL cells and the other arm with antigens on immune effector cells, facilitating direct contact between immune cells and CLL cells, leading to the lysis of the latter. Emerging BsAbs, exemplified by bispecific T cell involvement (BiTE), recruit T cells to tumor cells and promote the development of tumor immunotherapy [134]. For instance, CD3/CD19 BsAbs activate the immune-mediated killing of CLL cells by binding to CD19 on CLL cells and CD3 receptors on T cells [137]. Additionally, BsAbs can counteract the immunosuppressive effects of CLL by binding to immune checkpoint molecules, such as the PD-1/PD-L1 axis, thereby restoring the cytotoxic potential of T cells [138]. By elucidating the dual-targeting capabilities of BsAbs and their ability to redirect immune cells to cancer cells, we lay the groundwork for understanding their diverse clinical applications. The following section will delve into the clinical classification and therapeutic potential of these innovative agents.

### Types and characteristics of BsAbs in CLL therapy

With a solid understanding of the mechanisms that drive BsAb functionality, we now explore their clinical classification. Here, we examine how these mechanisms translate into distinct therapeutic categories, highlighting their potential to revolutionize cancer treatment. The FDA-approved CD3/CD19 BsAb, currently utilized for the treatment of ALL, has demonstrated potential therapeutic value against CLL. Its mechanism of action involves simultaneous binding to CD19-positive CLL cells and CD3-positive T cells, thereby harnessing the cytotoxic potential of T cells against CLL cells [139]. The CD3/CD20 BsAb engages CD20 on the surface of B cells and CD3 on the surface of T cells, enhancing T cell recognition and cytotoxicity against CLL cells. These antibodies, currently in trial phases, have exhibited exceptionally high clearance capabilities [140]. CD3/CD33 BsAb, in which one arm targets the CD3 molecule on the surface of T cells and the other arm targets the CD33 antigen on the surface of cancer cells, in this way, the BsAb close the distance between T cells and cancer cells, directing T cells to kill cancer cells directly [141]. The ROR1/CD3 BsAb capitalizes on the high expression of ROR1, a transmembrane protein, in CLL cells. By binding to ROR1 and CD3, this BsAb bridges T cells and CLL cells, enhancing T cell cytotoxicity against CLL cells [142]. (Table 3)

**Table 3:** Therapeutic applications of BsAb in CLL

Target	Drug name	Mechanism	Phase	Outcomes	Main indication	Antibody format
CD3/CD19	Blinatumomab	Bispecific binding enables T cells to aggregate around B cells; Activate T cells to release cytotoxic particles, thereby inducing cell death; Release cytokines to augment the immune response; Mediate the formation of synapses between T cells and B cells, thereby effectively transmitting cytotoxic signals.	II	Trend towards better OS; Better QOL.	R/R ALL, especially for patients who do not respond to traditional chemotherapy; MRD^+^ ALL.	BiTE
	AFM11	Recruit T cells to the vicinity of tumor cells and activate them; Facilitate the release of cytotoxic particles from T cells; Induce apoptosis via cytotoxic mechanisms; The immune-enhancing effect significantly exerts anti-tumor activity at lower drug concentrations.	-	-	R/R NHL; R/R ALL.	TandAb
CD3/CD20	Glofitamab	Activate T cells to release cytotoxic effector molecules, thereby inducing apoptosis; Activate the body’s immune system and induce the formation of immune memory; Cytokines released by T cells further enhance the immune response.	-	-	R/R DLBCL; R/R LBCL.	2:1 CrossMab
	Mosunetuzumab	Activate T cells to release cytotoxic proteins, which penetrate the B cell membrane, leading to the leakage of intracellular substances and ultimately inducing apoptosis; Enhance the activity of other immune cells to augment the immune response.	I/II	ORR 42.0% CR 23.9% PFS 3.2mos	R/R FL, specifically, adult patients who have undergone at least two systemic treatments; DLBCL.	Knobs-into-holes
	Odronextamab	Activating T cells leads to the release of cytotoxic particles, resulting in cytolytic cell death; Release cytokines to augment the immune system’s antitumor activity; Form immune memory to provide lasting immune protection; Alter the tumor microenvironment to reduce the number of immunosuppressive cells.	-	-	R/R FL and R/R DLBCL, specifically, adult patients who have received two or more systemic treatments.	Veloci-Bi platform
CD3/CD33	AMG330	Recruit T cells back to the vicinity of tumor cells and activate them; It forms immune synapses between T cells and tumor cells, thereby directly killing the tumor cells.	-	-	AML, especially R/R patients who do not respond to standard chemotherapy.	BiTE
	AMV564	Promote the expansion and activation of T cells to enhance the body’s immune response to tumors; T cells are recruited to the vicinity of tumor cells, where they form immune synapses, leading to tumor cell apoptosis; Selective depletion of MDSCs.	-	-	AML, especially those with high CD33 expression; MDS; solid tumors.	TandAb
CD3/ROR1	EMB−07	Activate T cells to release cytotoxic particles, thereby directly destroying tumor cells; T cells are recruited to the vicinity of tumor cells, where they form immune synapses to enhance their attack on the tumor cells; Reduce the release of cytokines to mitigate the risk of CRS.	I	Trend towards better OS; Better QOL.	Solid tumors; CLL; SLL; MCL; DLBCL.	MAT-Fab
	NVG−111	T cells are recruited to the vicinity of tumor cells, where they form immune synapses to enhance their attack on the tumor cells; Activate T cells to release cytotoxic particles, thereby directly destroying tumor cells; T cells release a variety of cytokines, thereby enhancing immune responses.	I/II	ORR 58% CR 100%	CLL, particularly R/R CLL; R/R MCL; NHL.	DART

ALL, Acute lymphoblastic leukemia; BsAb, Bispecific antibody; BiTE, Bispecific T cell engager; CLL, Chronic lymphocytic leukemia; CRS, Cytokine release syndrome; DART, Dual-affinity retargeting antibody; DLBCL, Diffuse large B-cell lymphoma; FL, Follicular lymphoma; LBCL, Large B-cell lymphoma; MAT-Fab, Monovalent Asymmetric Tandem Fab; MCL, Mantle cell lymphoma; MDS, Myelodysplastic syndromes; MDSCs, Myeloid-derived suppressor cells; MRD, Minimal residual disease; NHL, Non-Hodgkin lymphoma; ORR, Objective response rate; OS, Overall survival; PFS, Progression-free survival; QOL, Quality of life; TandAb, Tandem diabody; R/R, Relapsed/refractory

### Efficacy of BsAbs

The CD3-CD20 BsAb, a full-length, humanized IgG1 immunoglobulin molecule, is among the most widely utilized in the field. Its prototypical drug, Eporitamab (GEN3013), is known for its ability to recruit T-cell effector functions and has demonstrated robust clinical activity in patients with recurrent CD20 + B-cell NHL [143]. For example, a phase I/II clinical trial involving 45 patients with R/R B-cell NHL demonstrated that Eporitamab significantly induced T cell expansion, activation, and differentiation into Th1 and effector memory cells across all patient samples. The patient population had a median age of 65 years, with 60% being male and having received at least two prior lines of therapy. The study showed an ORR of 70% and a CR rate of 30%, with a median PFS of 12 months [144]. BsAb targeting CD20/CD3 T cells are capable of lysing CD20-expressing B cells, including primary leukemia and lymphoma cells, in both in vitro and in vivo settings [145]. Recent research, including clinical trials, has indicated that certain CD20 × CD3 BsAb exhibit significant anti-B-cell NHL activity by directing T cells to target CD20 + NHL cells [143]. Additionally, experimental data has demonstrated that these antibodies efficiently eliminate cancer cells in a dose-dependent manner in vitro within B lymphoblastic cells expressing CD20. These findings suggest that such antibodies hold potential clinical application value in the treatment of CD20-expressing B-cell malignancies [146].

### BsAbs combination therapy

The primary therapeutic strategy involves the combination of BsAbs with BTK inhibitors, such as ibrutinib. Ibrutinib as a monotherapy has demonstrated significant curative effects, and its combination with BsAbs has been shown to enhance these effects. For instance, a multicenter, open-label clinical trial involving 784 patients with R/R CLL demonstrated an ORR of 72.11% (95% CI: 66–77%; *p* < 0.0001) and a CR rate of 49.19% (95% CI: 39–59%; *p* > 0.05) when ibrutinib was combined with BsAbs. The patient population had a median age of 61 years, with nearly equal sex distribution. The study evaluated the efficacy of ibrutinib both as monotherapy and in combination with other agents [48]. The secondary option includes the combination with ICIs, such as PD-1/PD-L1 inhibitors. These inhibitors have shown considerable efficacy in overcoming the immunosuppression associated with CLL, and their combination with BsAbs may further augment treatment efficacy. A meta-analysis of 17 studies involving 514 patients with R/R diffuse large B-cell lymphoma (DLBCL) showed that ibrutinib-based combination therapies achieved a combined CR rate of 26% (95% CI: 0.19–0.34) and an ORR of 49% (95% CI: 0.40–0.58). The median PFS was 5.57 months (95% CI: 4.61–6.52) [147]. The tertiary option is the sequential use of CAR-T cell therapy and BsAbs. CAR-T cell therapy has demonstrated significant efficacy in CLL treatment, particularly in patients with relapsed or refractory disease. However, resistance to CAR-T therapy can emerge due to mechanisms such as antigen loss or immune evasion. In such cases, BsAbs targeting alternative antigens can be employed as a subsequent treatment strategy. This sequential approach leverages the initial efficacy of CAR-T cells while providing an additional therapeutic option when resistance develops. For example, in patients who experience relapse after CAR-T therapy due to CD19 loss, BsAbs targeting CD20 or other relevant antigens can be used to achieve sustained remission [148]. This strategy ensures that each therapy is utilized to its full potential, optimizing patient outcomes.

### Side effects and treatment of BsAbs

BsAb has demonstrated significant therapeutic benefits in the management of CLL, yet it is not without its adverse effects. The primary side effects associated with BsAb are CRS and ICANS. The incidence of CRS in BsAb therapy ranges from 20 to 50%, with the majority of cases being mild (grade 1–2) and only a minimal fraction being severe (grade 3–4) [149]. For example, a real-world study of patients treated with BsAbs reported that CRS occurred in 20–50% of cases, with grade 3–4 CRS affecting less than 10% of patients. ICANS, often occurring concurrently with or following CRS, was reported in approximately 10–20% of patients, with severe cases (grade 3–4) being rare [150]. As a precautionary measure against CRS, the prophylactic administration of corticosteroids is often advised, a strategy previously alluded to. The clinical presentation and preventative strategies for ICANS are analogous to those detailed earlier and thus will not be reiterated here. It is important to note that long-term adverse effects of BsAb therapy may include prolonged cytopenias and an increased risk of infections, which require vigilant monitoring and management [150].

### The advantages of BsAbs compared to other immunotherapies

BsAbs offer several therapeutic advantages over traditional monoclonal antibodies, particularly in the context of targeting multiple pathways involved in tumor progression. Initially, BsAbs targeting two or more pathways can potentially reduce drug resistance and the risk of tumor progression, as compared to monotherapy that targets a single pathway [151]. Subsidiarily, BsAbs can recruit immune cells to the TME and counteract the effects of immunosuppressive factors, thereby restoring anti-tumor immune activity [152]. Additionally, BsAbs can bridge T cells directly to tumor cells, activating T-cell-mediated cytotoxicity [153]. Compared to traditional monoclonal antibodies, BsAbs have a broader therapeutic window, enabling the achievement of therapeutic effects at lower doses and reducing side effects [154]. Furthermore, by simultaneously blocking two signaling pathways or linking two types of cells, BsAbs can enhance therapeutic efficacy while minimizing systemic side effects, striking a balance between efficacy and safety [153]. Lastly, BsAbs’ ability to bind two different antigens or two epitopes on the same antigen provides more precise targeting capabilities, which can enhance the therapeutic effect and reduce off-target damage to normal cells [155].

In conclusion, BsAbs hold significant potential in the therapeutic landscape of CLL. Nevertheless, the journey towards continuous program optimization, side effect reduction, and the development of novel drugs is an extensive one. The production of BsAbs is highly complex due to their unique structure and the need for specialized processes to ensure high yield and quality. However, innovative production technologies, such as advanced protein engineering and purification techniques, are being developed to optimize BsAbs' therapeutic potential and reduce production costs. These innovations not only enhance the efficiency of BsAbs manufacturing but also improve their pharmacokinetics and efficacy. Collaboration with regulatory agencies is crucial in this process. For example, the China-UK drug regulatory cooperation project has identified areas of mutual interest to push forward technical exchanges and deepen cooperation. This collaboration can facilitate the approval process and ensure that BsAbs meet regulatory standards, thereby enhancing their accessibility. Innovative pricing and reimbursement strategies are also essential to reduce the economic burden on patients. By working with regulatory bodies and adopting new production technologies, the biopharmaceutical industry can make BsAbs more affordable and accessible, while also exploring new therapeutic possibilities. Future directions may include further optimization of BsAbs' half-life and efficacy, as well as the development of new formats and structures to target a wider range of diseases. Despite these challenges, with persistent research efforts, BsAbs are anticipated to become a vital component of CLL treatment, offering improved outcomes for patients.

### The next generation therapy of BsAbs - TsAbs

The evolution of BsAbs has led to the conception of tri-specific antibodies (TsAbs), which incorporate a co-stimulatory protein or an additional targeted antigen to amplify the efficacy of the antibody response. While TsAbs represent a step forward in antibody engineering, they are not without their challenges and require substantial future development. TsAbs are designed to target three distinct antigen epitopes on tumor cells and immune effector cells, thereby enhancing therapeutic efficacy and safety [156]. This design allows for the simultaneous engagement of multiple immune cells, improving the specificity and potency of the immune response against cancer cells. In preclinical studies, TsAbs have demonstrated remarkable efficiency in overcoming immune escape and significantly enhancing antitumor efficacy [157]. For instance, a CD19/CD22/CD3 TsAb has shown superior performance in inducing T-cell-specific cytotoxicity and cytokine production against B-cell malignancies, compared to other formats [158]. This innovative approach holds great promise for the future of cancer immunotherapy.

Despite the enormous potential of TsAbs in cancer immunotherapy, its clinical application faces significant regulatory challenges, particularly in balancing safety and efficacy. TsAbs can simultaneously target three distinct antigens, thereby potentially enhancing antitumor activity. However, this complexity also increases the risk of adverse events such as CRS, a severe inflammatory response triggered by the overactivation of immune cells. Ensuring safety requires rigorous preclinical testing and phased clinical trials to monitor and mitigate such risks. Additionally, the intricate structure of TsAbs may lead to immunogenicity, causing the production of anti-drug antibodies (ADAs) that could neutralize the therapeutic effects. Regulatory bodies must therefore establish stringent guidelines to ensure the thorough evaluation of these novel agents, balancing their therapeutic potential with the need to safeguard patients from potential harm.

## Antibody drug conjugate therapy

### Mechanism of ADCs

ADCs represent a significant class of oncology therapeutics, comprising a mAb conjugated to a cytotoxic drug, also known as a payload, through a linker molecule [159]. These conjugates are designed to harness the high specificity targeting ability of mAbs and the potent cytotoxic effects of small-molecule drugs, thereby achieving precise and efficacious elimination of cancer cells [160, 161]. ADCs are typically structured with mAbs covalently linked to cytotoxic agents via chemical linkers [162]. They leverage the antigen-recognition capabilities of antibodies to selectively deliver cytotoxic drugs to tumor cells, with the linker ensuring the stability and release of the payload at the tumor site [163]. Beyond direct cytotoxicity, the mechanisms of action of ADCs encompass the induction of anti-tumor immune responses and the inhibition of downstream signal transduction pathways associated with antigen receptors [164].

### Application of ADCs in leukemia

Brentuximab Vedotin (BV) marks the first ADC approved for the treatment of lymphomas, specifically targeting CD30 [165, 166], an antigen robustly expressed in hematologic tumors, particularly HL [167]. BV binds to CD30 on the tumor surface and facilitates the internal release of toxins through lysosomal degradation, thereby inducing tumor cell death [168]. Polatuzumab Vedotin (PV) is another ADC that targets CD79b on B-cell tumor surfaces, sharing a similar mechanism of action to BV [169, 170]. Loncastuximab Tesirine is a CD19-targeting ADC, derived from a humanized CD19 monoclonal antibody linked to a dimer toxin via a linker [171, 172]. It is primarily indicated for the treatment of R/R DLBCLthat has been previously treated with two or more systemic therapies [173, 174]. Gemtuzumab Ozogamicin, a CD33-targeting ADC initially approved in 2000 and re-approved after a market withdrawal, is primarily utilized for the treatment of AML [175, 176]. It has demonstrated significant improvements in patient survival and exhibits fewer adverse reactions compared to other therapies [177]. Inotuzumab Ozogamicin, an ADC targeting CD22 [178], is predominantly used for the treatment of lymphatic leukemia, especially R/R ALL [179]. Moxetumomab Pasudotox, which has been recently marketed, is developed for the treatment of R/R Hairy Cell Leukemia (HCL) which has failed to respond to more than two prior treatments, with CD22 as its primary target [180]. Studies have indicated that its treatment of HCL is more stable and less toxic compared to other drugs [181]. Lastly, Belantamab Mafodotin, an ADC drug representative for myeloma, is used for the treatment of adult patients with refractory MM who have received at least four prior lines of treatment, including a CD38 monoclonal antibody, a proteasome inhibitor, and an immunomodulatory agent [182]. However, it was withdrawn from the market in 2022 due to insufficient survival benefits compared to conventional therapies and the presence of fatal side effects [183]. (Table 4)

**Table 4:** The progress of ADCs in the treatment of CLL

Drug name	Target	Mechanism	FDA approval time	Main indication
Brentuximab Vedotin	CD30	The drug is internalized into lysosomes by cells, where it releases MMAE; As a tubulin inhibitor, MMAE binds to peripherins within cells, disrupting the microtubule network and inducing apoptosis; The released MMAE diffuses into the surrounding tumor microenvironment, killing tumor cells in a phenomenon known as the bystander effect.	2011	CHL, SALCL, PCALCL, PTCL, MF.
Polatuzumab Vedotin	CD79b	Mechanism of action of Brentuximab Vedotin; Through its ADC mechanism, it directly kills CLL cells, particularly those that are resistant to traditional chemotherapy.	2019	R/R DLBCL, CLL.
Loncastuximab Tesirine	CD19	The drug is internalized into the cell, where intracellular enzymes hydrolyze the linker to release the PBD cytotoxin; The released PBD disrupts normal DNA metabolism, leading to the arrest of DNA replication and the induction of apoptosis.	2021	R/R DLBCL, CLL.
Gemtuzumab Ozogamicin	CD33	The compound is internalized by cells into the acidic lysosomal environment, where the linker undergoes hydrolysis to release the potent cytotoxic agent, calicheamicin; Calicheamicin binds to DNA, inducing double-strand breaks that lead to cell death.	2017	AML.
Inotuzumab Ozogamicin	CD22	Upon cellular uptake, the linker is hydrolyzed within the acidic environment, leading to the release of the small molecule drug N-acetyl-γ-calicheamicin dimethylhydrazide; The released drug binds to DNA, causing double-strand breaks and inducing apoptosis.	2017	R/R ALL.
Moxetumomab Pasudotox	CD22	Upon cellular internalization, pseudomonas aeruginosa exotoxin A is released into the intracellular environment; Once the toxin enters the cell, it inhibits protein synthesis; The inhibition of protein synthesis prevents cells from growing and proliferating, ultimately leading to apoptosis.	2018	R/R HCL, CLL.

ADC, Antibody-drug conjugate; AML, Acute myeloid leukemia; ALL, Acute lymphoblastic leukemia; CHL, Classical hodgkin lymphoma; CLL, Chronic lymphocytic leukemia; DLBCL, Diffuse large B-cell lymphoma; HCL, Hairy cell leukemia; MF, Mycosis fungoides; MMAE, Monomethyl auristatin E; PBD, Pyrrolobenzodiazepine dimer; PCALCL, Primary cutaneous anaplastic large cell lymphoma; PTCL, Peripheral T-cell lymphomas; SALCL, Systematic anaplastic large cell lymphoma; R/R, Relapsed/refractory

### ADCs combination therapy

The therapeutic potential of ADCs can be augmented through combination regimens with targeted drugs such as BCL-2 inhibitors, which inhibit the anti-apoptotic pathways of CLL cells, thereby potentially enhancing the cytotoxic effects of ADCs. Additionally, the combination of ADCs with ICIs aims to stimulate the immune system, further augmenting the cytotoxic effects on CLL cells. ADCs are also combined with chemotherapeutic drugs to enhance therapeutic efficacy and mitigate drug resistance. For instance, the combination of ADCs with BTK inhibitors targets cell signal transduction pathways, thereby potentially enhancing the cytotoxic effects of ADCs.

ADCs exhibit potential in the treatment of CLL, but several challenges remain to be addressed. Currently, the application of ADCs in CLL is still in the early stages of development, with limited clinical data available. However, some ADCs have shown promising preclinical and early-phase clinical results. For example, PV has demonstrated an ORR of 40% and a median PFS of 6 months in a Phase I/II study involving patients with R/R CLL. Another ADC, Inotuzumab ozogamicin, has also shown potential in CLL, although its use is more commonly associated with ALL. Despite these encouraging results, challenges such as drug resistance, tumor heterogeneity, and adverse reactions need to be addressed. Future research will focus on further optimizing ADC design and exploring novel targets and combination therapy regimens to minimize the incidence of adverse reactions. As an innovative therapeutic approach, ADCs are anticipated to offer promising advancements for CLL patients, in tandem with the ongoing advancements in science and technology.

## γδ T cell therapy

### Introduction of γδ T cell

In the realm of cell therapy, specifically within CAR-T cell therapy, the majority of T cells utilized are αβ T cells. These cells express TCRs composed of two heterodimeric peptide chains, α and β, which constitute approximately 95% of the T cell population [184]. Additionally, a minor subset of T cells is composed of γ and δ chains, termed γδ T cells, which represent about 0.5–5% of the total T cell population. γδ T cells possess both innate and adaptive immune response characteristics, enabling them to rapidly recognize non-MHC-restricted tumor antigens and mount an immune response accordingly [185, 186].

### Mechanism of γδ T cell therapy

Gentles et al. conducted a comprehensive analysis of gene expression profiles from approximately 18,000 samples across 39 different types of malignant tumors and identified a significant correlation between the presence of tumor-infiltrating γδ T cells and favorable prognosis [187]. Currently, the potential of γδ T cells in CAR-based immunotherapies for cancer has been substantiated. Beyond the CAR-mediated cytotoxicity, γδ T cells can recognize tumor cells through their intrinsic receptors, including Vδ1 TCR, CD16, NKG2D, and NKp30, and activate various natural tumor-killing pathways. These pathways encompass perforin/granzyme-dependent cytotoxicity, CD16-mediated ADCC, and Tumor Necrosis Factor-Related Apoptosis-Inducing Ligand (TRAIL)/ Fas Ligand (FASL)-induced apoptosis [188]. Furthermore, γδ T cells can function as antigen-presenting cells (APCs), presenting tumor antigens to αβ T cells. Studies have demonstrated that CAR-γδ T cells, engineered to target GD2, not only specifically eliminate GD2-positive LAN1 cells but also maintain their capacity to phagocytose and present tumor antigens to αβ T cells, thereby inducing the clonal expansion of αβ T cells [189].γδ T cells exhibit a superior homing capability, which allows them to more effectively infiltrate tumor tissues, particularly those characterized by a hypoxic TME. Their efficacy within a hypoxic TME is further enhanced because their cytotoxic properties and the secretion of key cytokines and chemokines, such as macrophage inflammatory protein MIP-1, tumor necrosis factor RANTES, and tumor necrosis factor-related activating protein CD40L, can be augmented under hypoxic conditions. Studies have demonstrated that engineering γδT cells with CAR can amplify their cytotoxic potential while preserving their migratory capacity to tumor cells and their ability to cross-present antigens. This modification represents a promising strategy to bolster the anti-tumor immune response [189].

### γδ T cell combination therapy

Combination therapy strategies are pivotal in augmenting the efficacy of γδ T cell therapy. One such strategy involves the integration with chemotherapy, which can disrupt the TME, thereby potentially enhancing the infiltration and activity of γδ T cells. Evidence suggests that the combination of γδ T cell therapy with chemotherapy can lead to improved therapeutic outcomes. Another approach is the combination with radiotherapy, which can stimulate the immune response against the tumor and enhance the anti-tumor activity of γδ T cells. The synergy between γδ T cells and ICI therapy can alleviate tumor-mediated immunosuppression, producing a cooperative effect that enhances therapeutic efficacy. When combined with BsAbs, γδ T cells can be directed more effectively to the tumor site, augmenting their cytotoxic capabilities. Lastly, the combination of CAR-γδ T cells with conventional γδ T cells may offer additional therapeutic benefits [190].

### Future research direction of γδ T cell combination therapy

Initially, it is crucial to explore the optimal combination of γδ T cell therapy with other treatment modalities to maximize therapeutic efficacy. Subsequently, there is a need for a greater number of large-scale clinical trials to verify the efficacy and safety of γδ T cell therapy in the context of CLL treatment. Additionally, the development of a novel evaluation system is essential for more precisely identifying patients who are suitable candidates for γδ T cell therapy and for monitoring their progress in real time [190]. Ultimately, further research should focus on elucidating the mechanisms of action of γδ T cells within the TME and investigating how their anti-tumor activity can be enhanced through technical interventions.

γδ T cell therapy introduces a novel therapeutic strategy for the treatment of CLL. Although the current state of the technology is not fully mature, preliminary results suggest that this therapy may emerge as a significant treatment option for CLL. Future research will necessitate multi-level studies to fully harness the potential of γδ T cells and to optimize their role in CLL treatment.

## Biological response modifier activated killer therapy

### Mechanism of BAK therapy

Biological Response Modifier (BRM)-Activated killer therapy, abbreviated as BAK therapy [191], is a form of immunotherapy that involves the extraction of immune cells from a patient’s body, their subsequent in vitro cultivation, proliferation, and activation, followed by their reintroduction into the patient via intravenous infusion [192]. This therapy aims to enhance the accuracy of cancer cell identification by the electrified immune cells, thereby minimizing the attack on both normal cells and cancer cells, leading to a characteristic minimal side effects profile [193]. BAK therapy predominantly utilizes non-MHC restricted lymphocytes, specifically CD56 + cells, which include gamma delta T cells and NK cells.

### Efficacy of BAK therapy

Researchers have successfully employed BAK therapy in the treatment of abdominal tumors, demonstrating its efficacy by showing that locally administered BAK lymphocytes can penetrate cancerous tissue and effectively eliminate cancer cells [194]. This finding is supported by evidence indicating that BAK therapy, utilizing CD56-positive lymphocytes, is effective against advanced solid cancers. Hospital-based researchers have further expanded the application of this therapy to liver cancer by administering BAK cells intravenously, thereby establishing a foundation for its use in hepatic malignancy treatment [195].

BAK therapy is advantageous in that it can be conducted on an outpatient basis, involving only blood collection and infusion, which eliminates the need for hospitalization. This therapy is also notable for its compatibility with other treatment modalities, such as radiation therapy, and has been increasingly integrated into clinical practice [196]. Primarily aimed at treating solid tumors, BAK therapy is also recognized for its potential to prevent cancer recurrence, offering anticipated high preventive efficacy.

However, at present, BAK therapy is not yet considered a standard treatment for leukemia and other hematological malignancies, despite previous research proposals suggesting its potential utility [197]. Due to its reliance on autologous immunity, BAK therapy has been recognized as a promising "fourth cancer treatment modality" and has garnered significant attention in recent years. Characterized by a favorable safety profile with minimal side effects, BAK therapy is a subject of high anticipation for future research and development.

## Conclusion

In recent years, advancements in science and technology have ushered in fundamental changes in the therapeutic landscape for CLL. The transition from chemotherapeutic immunotherapy of the last century to the current first-line targeted therapies and the rapidly evolving field of cell therapy has significantly altered patient prognoses. Currently, CD20 mAbs and CAR cell therapy are widely employed in the clinical treatment of CLL. Other immunotherapeutic approaches, such as BsAbs, ADCs, and γδ T cell therapy, show great promise; however, the challenges in their development should not be overlooked. For CLL patients, the development of the most rational personalized treatment plans, based on genetic profiling and risk assessment, is essential. Additionally, exploring fresh targeted drugs in combination with immunotherapies to enhance therapeutic outcomes is a critical direction for research. While novel treatments offer improved response rates and survival, the majority of CLL patients eventually experience relapse. Consequently, scientists have proposed the use of Allo-HSCT as a potential emerging avenue for CLL treatment, which may present advanced opportunities for therapeutic breakthroughs.

## Data Availability

No datasets were generated or analysed during the current study.

## Abbreviations

AAV: ANCA-Associatedvasculitis
ACT: Adoptive Cell Therapy
ADAs: Anti-Drug Antibodies
ADCC: Antibody-Dependent Cellular Cytotoxicity
ADCs: Antibody-Drug Conjugates
ALCL: Anaplastic Large Cell Lymphoma
ALL: Acute Lymphoblastic Leukemia
Allo-HSCT: Allogeneic Hematopoietic Stem Cell Transplantation
AML: Acute Myeloid Leukemia
ANCA: Antineutrophil Cytoplasmic Antibodies
APCs: Antigen-Presenting Cells
BAK: BRM Activated Killer
BCL: B-Cell Lymphoma
BiTE: Bispecific T Cell Engager
BRM: Biological Response Modifier
BsAb: Bispecific Antibody
BsAbs: Bispecific Antibodies
BTK: Bruton’s Tyrosine Kinase
BV: Brentuximab Vedotin
CAR: Chimeric Antigen Receptor
CAR-Ms: Car-Macrophages
CDC: Complement-Dependent Cytotoxicity
CHL: Classical Hodgkin Lymphoma
CIT: Chemoimmunotherapy
CLL: Chronic Lymphocytic Leukemia
CR: Complete Response
CRS: Cytokine Release Syndrome
DART: Dual-Affinity Retargeting Antibody
DLBCL: Diffuse Large B-cell Lymphoma
FASL: Fas Ligand
Fc: Fragment Crystallizable
FCR: Fludarabine, Cyclophosphamide, Rituximab
FL: Follicular Lymphoma
GvHD: Graft-Versus-Host Disease
HCL: Hairy Cell Leukemia
HL: Hodgkin’s Lymphoma
ICANS: Immune Effector Cell-Associated Neurotoxicity Syndrome
ICIs: Immune Checkpoint Inhibitors
IFN-γ: Interferon-γ
IL-2: Interleukin-2
IL-6: Interleukin-6
iPSCs: induced Pluripotent Stem Cells
irAEs: immune-Related Adverse Events
ITP: Immune Thrombocytopenia
LBCL: Large B-cell Lymphoma
mAb: monoclonal Antibody
mAbs: monoclonal Antibodies
MAC: Membrane attack Complex
MAT-Fab: Monovalent Asymmetric Tandem Fab
MCL: Mantle Cell Lymphoma
MDS: Myelodysplastic Syndromes
MDSCs: Myeloid-Derived Suppressor Cells
MDSCs: Myeloid-Derived Suppressor Cells
MF: Mycosis Fungoides
MHC: Major Histocompatibility Complex
MM: Multiple Myeloma
MMAE: Monomethyl Auristatin E
MRD: Minimal Residual Disease
MS: Multiple Sclerosis
NHL: Non-Hodgkin Lymphoma
NK: Natural Killer
ORR: Objective Response Rate
OS: Overall Survival
PBD: Pyrrolobenzodiazepine Dimer
PCALCL: Primary Cutaneous Anaplastic Large Cell Lymphoma
PD-1: Programmed Death-1
PD-L1: Programmed Death-Ligand 1
PFS: Progression-Free Survival
PR: Partial Response Rate
PTCL: Peripheral T-cell Lymphomas
PV: Polatuzumab Vedotin
QOL: Quality of Life
R/R: Relapsed/Refractory
RA: Rheumatoid Arthritis
RMS: Relapsed Multiple Sclerosis
RT: Richter’s Transformation
SALCL: Systematic Anaplastic Large Cell Lymphoma
SLE: Systemic Lupus Erythematosus
SLL: Small Lymphocytic Lymphoma
TAMs: Tumor-Associated Macrophages
TAMs: Tumor-Associated Macrophages
TandAb: Tandem Diabody
TCRs: T cell Receptors
TLS: Tumor Lysis Syndrome
TME: Tumor Microenvironment
TNF-α: Tumor Necrosis Factor-α
TRAIL: Tumor Necrosis Factor-Related Apoptosis-Inducing Ligand
Tregs: Regulatory T Cells
TRUCKs: T cells Redirected for Universal Cytokine Killing
TsAbs: Trispecific Antibodies
Ven-R: Venetoclax-Rituximab

## References

[1] Hallek M, Shanafelt TD, Eichhorst B. Chronic lymphocytic leukaemia. Lancet. 2018;391:1524–37. 29477250 10.1016/S0140-6736(18)30422-7

[2] Zhang X, Han Y, Hu X, Wang H, Tian Z, Zhang Y, et al. Competing endogenous RNA networks related to prognosis in chronic lymphocytic leukemia: comprehensive analyses and construction of a novel risk score model. Biomark Res. 2022;10:75. 36271413 10.1186/s40364-022-00423-y PMC9585723

[3] Montserrat E, Bosch F, Rozman C. B-cell chronic lymphocytic leukemia: recent progress in biology, diagnosis, and therapy. Ann Oncol. 1997;8(Suppl 1):93–101. 9187440

[4] Bosch F, Dalla-Favera R. Chronic lymphocytic leukaemia: from genetics to treatment. Nat Rev Clin Oncol. 2019;16:684–701. 31278397 10.1038/s41571-019-0239-8

[5] Woyach JA. Management of relapsed/refractory chronic lymphocytic leukemia. Am J Hematol. 2022;97(Suppl 2):S11–8. 36125037 10.1002/ajh.26683 PMC9826056

[6] Cheson BD, Sharman JP. Current approaches and novel new agents in the treatment of chronic lymphocytic leukemia. JCO Oncol Pract. 2024;OP2300770. 10.1200/OP.23.00770 38848511

[7] Bennett R, Seymour JF. Update on the management of relapsed/refractory chronic lymphocytic leukemia. Blood Cancer J. 2024;14:33. 38378673 10.1038/s41408-024-01001-1 PMC10879527

[8] Visentin A, Frazzetto S, Trentin L, Chiarenza A. Innovative combinations, cellular therapies and bispecific antibodies for chronic lymphocytic leukemia: A narrative review. Cancers (Basel). 2024;16:1290. 38610967 10.3390/cancers16071290 PMC11011076

[9] Frampton JE, Wagstaff AJ, Alemtuzumab. Drugs. 2003;63:1229–43. discussion 1245–1246. 12790693 10.2165/00003495-200363120-00003

[10] Hallek M, Pflug N. Chronic lymphocytic leukemia. Ann Oncol. 2010;21(Suppl 7):vii154–164. 20943609 10.1093/annonc/mdq373

[11] Rougé L, Chiang N, Steffek M, Kugel C, Croll TI, Tam C, et al. Structure of CD20 in complex with the therapeutic monoclonal antibody rituximab. Science. 2020;367:1224–30. 32079680 10.1126/science.aaz9356

[12] Maloney DG, Smith B, Rose A. Rituximab: mechanism of action and resistance. Semin Oncol. 2002;29:2–9. 28140087 10.1053/sonc.2002.30156

[13] Tedder TF, Streuli M, Schlossman SF, Saito H. Isolation and structure of a cDNA encoding the B1 (CD20) cell-surface antigen of human B lymphocytes. Proc Natl Acad Sci U S A. 1988;85:208–12. 2448768 10.1073/pnas.85.1.208 PMC279513

[14] Smith MR. Rituximab (monoclonal anti-CD20 antibody): mechanisms of action and resistance. Oncogene. 2003;22:7359–68. 14576843 10.1038/sj.onc.1206939

[15] Teeling JL, French RR, Cragg MS, van den Brakel J, Pluyter M, Huang H, et al. Characterization of new human CD20 monoclonal antibodies with potent cytolytic activity against non-Hodgkin lymphomas. Blood. 2004;104:1793–800. 15172969 10.1182/blood-2004-01-0039

[16] Browning JL. B cells move to centre stage: novel opportunities for autoimmune disease treatment. Nat Rev Drug Discov. 2006;5:564–76. 16816838 10.1038/nrd2085

[17] Maddocks KJ, Lin TS. Update in the management of chronic lymphocytic leukemia. J Hematol Oncol. 2009;2:29. 19619273 10.1186/1756-8722-2-29 PMC2723130

[18] Brown JR, Cymbalista F, Sharman J, Jacobs I, Nava-Parada P, Mato A. The role of rituximab in chronic lymphocytic leukemia treatment and the potential utility of biosimilars. Oncologist. 2018;23:288–96. 29212732 10.1634/theoncologist.2017-0150 PMC5905689

[19] Saini KS, Azim HA, Cocorocchio E, Vanazzi A, Saini ML, Raviele PR, et al. Rituximab in hodgkin lymphoma: is the target always a hit? Cancer Treat Rev. 2011;37:385–90. 21183282 10.1016/j.ctrv.2010.11.005

[20] Coiffier B, Lepage E, Briere J, Herbrecht R, Tilly H, Bouabdallah R, et al. CHOP chemotherapy plus rituximab compared with CHOP alone in elderly patients with diffuse large-B-cell lymphoma. N Engl J Med. 2002;346:235–42. 11807147 10.1056/NEJMoa011795

[21] Czuczman MS, Trněný M, Davies A, Rule S, Linton KM, Wagner-Johnston N, et al. A phase 2/3 multicenter, randomized, Open-Label study to compare the efficacy and safety of Lenalidomide versus investigator’s choice in patients with relapsed or refractory diffuse large B-Cell lymphoma. Clin Cancer Res. 2017;23:4127–37. 28381416 10.1158/1078-0432.CCR-16-2818 PMC8171498

[22] Wu M, Akinleye A, Zhu X. Novel agents for chronic lymphocytic leukemia. J Hematol Oncol. 2013;6:36. 23680477 10.1186/1756-8722-6-36 PMC3659027

[23] Byrd JC, Kipps TJ, Flinn IW, Castro J, Lin TS, Wierda W, et al. Phase 1/2 study of lumiliximab combined with fludarabine, cyclophosphamide, and rituximab in patients with relapsed or refractory chronic lymphocytic leukemia. Blood. 2010;115:489–95. 19843887 10.1182/blood-2009-08-237727 PMC2810983

[24] Cohen JI. Clinical practice: herpes Zoster. N Engl J Med. 2013;369:255–63. 23863052 10.1056/NEJMcp1302674 PMC4789101

[25] Wei G, Wang J, Huang H, Zhao Y. Novel immunotherapies for adult patients with B-lineage acute lymphoblastic leukemia. J Hematol Oncol. 2017;10:150. 28821272 10.1186/s13045-017-0516-x PMC5563021

[26] Elias S, Kahlon S, Kotzur R, Kaynan N, Mandelboim O. Obinutuzumab activates FcγRI more potently than other anti-CD20 antibodies in chronic lymphocytic leukemia (CLL). Oncoimmunology. 2018;7:e1428158. 29872553 10.1080/2162402X.2018.1428158 PMC5980409

[27] Golay J, Da Roit F, Bologna L, Ferrara C, Leusen JH, Rambaldi A, et al. Glycoengineered CD20 antibody obinutuzumab activates neutrophils and mediates phagocytosis through CD16B more efficiently than rituximab. Blood. 2013;122:3482–91. 24106207 10.1182/blood-2013-05-504043

[28] Morschhauser FA, Cartron G, Thieblemont C, Solal-Céligny P, Haioun C, Bouabdallah R, et al. Obinutuzumab (GA101) monotherapy in relapsed/refractory diffuse large b-cell lymphoma or mantle-cell lymphoma: results from the phase II GAUGUIN study. J Clin Oncol. 2013;31:2912–9. 23835718 10.1200/JCO.2012.46.9585

[29] Randen U, Trøen G, Tierens A, Steen C, Warsame A, Beiske K, et al. Primary cold agglutinin-associated lymphoproliferative disease: a B-cell lymphoma of the bone marrow distinct from lymphoplasmacytic lymphoma. Haematologica. 2014;99:497–504. 24143001 10.3324/haematol.2013.091702 PMC3943313

[30] Hillmen P, Pitchford A, Bloor A, Broom A, Young M, Kennedy B, et al. Ibrutinib and rituximab versus fludarabine, cyclophosphamide, and rituximab for patients with previously untreated chronic lymphocytic leukaemia (FLAIR): interim analysis of a multicentre, open-label, randomised, phase 3 trial. Lancet Oncol. 2023;24:535–52. 37142374 10.1016/S1470-2045(23)00144-4

[31] Shanafelt TD, Wang XV, Kay NE, Hanson CA, O’Brien S, Barrientos J, et al. Ibrutinib-Rituximab or chemoimmunotherapy for chronic lymphocytic leukemia. N Engl J Med. 2019;381:432–43. 31365801 10.1056/NEJMoa1817073 PMC6908306

[32] Shanafelt TD, Wang XV, Hanson CA, Paietta EM, O’Brien S, Barrientos J, et al. Long-term outcomes for ibrutinib-rituximab and chemoimmunotherapy in CLL: updated results of the E1912 trial. Blood. 2022;140:112–20. 35427411 10.1182/blood.2021014960 PMC9283968

[33] Woyach JA, Ruppert AS, Heerema NA, Zhao W, Booth AM, Ding W, et al. Ibrutinib regimens versus chemoimmunotherapy in older patients with untreated CLL. N Engl J Med. 2018;379:2517–28. 30501481 10.1056/NEJMoa1812836 PMC6325637

[34] Lipsky A, Martin P. Bendamustine-rituximab in mantle cell lymphoma. Lancet Haematol. 2017;4:e2–3. 27927585 10.1016/S2352-3026(16)30187-9

[35] Byrd JC, Harrington B, O’Brien S, Jones JA, Schuh A, Devereux S, et al. Acalabrutinib (ACP-196) in relapsed chronic lymphocytic leukemia. N Engl J Med. 2016;374:323–32. 26641137 10.1056/NEJMoa1509981 PMC4862586

[36] Eichhorst B, Fink A-M, Bahlo J, Busch R, Kovacs G, Maurer C, et al. First-line chemoimmunotherapy with Bendamustine and rituximab versus fludarabine, cyclophosphamide, and rituximab in patients with advanced chronic lymphocytic leukaemia (CLL10): an international, open-label, randomised, phase 3, non-inferiority trial. Lancet Oncol. 2016;17:928–42. 27216274 10.1016/S1470-2045(16)30051-1

[37] Kater AP, Wu JQ, Kipps T, Eichhorst B, Hillmen P, D’Rozario J, et al. Venetoclax plus rituximab in relapsed chronic lymphocytic leukemia: 4-Year results and evaluation of impact of genomic complexity and gene mutations from the MURANO phase III study. J Clin Oncol. 2020;38:4042–54. 32986498 10.1200/JCO.20.00948 PMC7768340

[38] Zinzani PL, Flinn IW, Yuen SLS, Topp MS, Rusconi C, Fleury I, et al. Venetoclax-rituximab with or without Bendamustine vs Bendamustine-rituximab in relapsed/refractory follicular lymphoma. Blood. 2020;136:2628–37. 32785666 10.1182/blood.2020005588 PMC7735159

[39] Papazoglou D, Wang XV, Shanafelt TD, Lesnick CE, Ioannou N, De Rossi G, et al. Ibrutinib-based therapy reinvigorates CD8 + T cells compared to chemoimmunotherapy: immune monitoring from the E1912 trial. Blood. 2024;143:57–63. 37824808 10.1182/blood.2023020554 PMC10797553

[40] Salles G, Barrett M, Foà R, Maurer J, O’Brien S, Valente N, et al. Rituximab in B-Cell hematologic malignancies: A review of 20 years of clinical experience. Adv Ther. 2017;34:2232–73. 28983798 10.1007/s12325-017-0612-x PMC5656728

[41] Byrd JC, Peterson BL, Morrison VA, Park K, Jacobson R, Hoke E, et al. Randomized phase 2 study of fludarabine with concurrent versus sequential treatment with rituximab in symptomatic, untreated patients with B-cell chronic lymphocytic leukemia: results from cancer and leukemia group B 9712 (CALGB 9712). Blood. 2003;101:6–14. 12393429 10.1182/blood-2002-04-1258

[42] James PA, Hartz AJ, Levy BT. Specialty of ambulatory care physicians and mortality after myocardial infarction. N Engl J Med. 2003;348:1288–9. author reply 1288–1289. 12660397 10.1056/NEJM200303273481316

[43] Hallek M, Chronic Lymphocytic L. 2025 Update on the epidemiology, pathogenesis, diagnosis, and therapy. Am J Hematol. 2025;100:450–80. 39871707 10.1002/ajh.27546 PMC11803567

[44] Davis TA, Grillo-López AJ, White CA, McLaughlin P, Czuczman MS, Link BK, et al. Rituximab anti-CD20 monoclonal antibody therapy in non-Hodgkin’s lymphoma: safety and efficacy of re-treatment. J Clin Oncol. 2000;18:3135–43. 10963642 10.1200/JCO.2000.18.17.3135

[45] Obeng EA, Carlson LM, Gutman DM, Harrington WJ, Lee KP, Boise LH. Proteasome inhibitors induce a terminal unfolded protein response in multiple myeloma cells. Blood. 2006;107:4907–16. 16507771 10.1182/blood-2005-08-3531 PMC1895817

[46] Ng AP, Worth L, Chen L, Seymour JF, Prince HM, Slavin M, et al. Cytomegalovirus dnaemia and disease: incidence, natural history and management in settings other than allogeneic stem cell transplantation. Haematologica. 2005;90:1672–9. 16330442

[47] Thouenon R, Verdeil G. Tumor microenvironment squeezes out the juice from T cells. Cell Res. 2024;34(10):677-678. 10.1038/s41422-024-00987-4 PMC11443050 38858609

[48] Arseni L, Sigismondo G, Yazdanparast H, et al. Longitudinal omics data and preclinical treatment suggest the proteasome inhibitor carfilzomib as therapy for ibrutinib-resistant CLL. Nat Commun. 2025;16(1):1041. 10.1038/s41467-025-56318-7 PMC11762753 39863584

[49] Knickmeier C, Noubissi Nzeteu GA, Gibbs BF, et al. It's about TIME - Gal-9 as a potential immunotherapeutic target in pancreatic ductal adenocarcinoma. Front Immunol. 2025;16:1495907. 10.3389/fimmu.2025.1495907 PMC11825744 39958335

[50] Zhao X, Liu B, William WN, et al. Interferon-ε loss is elusive 9p21 link to immune-cold tumors, resistant to immune-checkpoint therapy and endogenous CXCL9/10 induction. J Thorac Oncol. 2024. 10.1016/j.jtho.2024.12.020 PMC13460200 39725169

[51] Floudas CS, Goswami M, Donahue RN, Pastor DM, Redman JM, Brownell I et al. Novel combination immunotherapy and clinical activity in patients with HPV-Associated cancers: A nonrandomized clinical trial. JAMA Oncol. 2025. 10.1001/jamaoncol.2024.6998 PMC11843463 39976981

[52] Clements AN, Casillas AL, Flores CE, et al. Inhibition of PIM kinase in tumor-associated macrophages suppresses inflammasome activation and sensitizes prostate cancer to immunotherapy. Cancer Immunol Res. 2025. 10.1158/2326-6066.CIR-24-0591 PMC12048269 39982419

[53] Robak T. Rituximab for chronic lymphocytic leukemia. Expert Opin Biol Ther. 2012;12:503–15. 22369284 10.1517/14712598.2012.665444

[54] Luo C, Wu G, Huang X, Ma Y, Zhang Y, Song Q, et al. Efficacy and safety of new anti-CD20 monoclonal antibodies versus rituximab for induction therapy of CD20 + B-cell non-Hodgkin lymphomas: a systematic review and meta-analysis. Sci Rep. 2021;11:3255. 33547368 10.1038/s41598-021-82841-w PMC7864901

[55] Song Y, Gao Q, Zhang H, Fan L, Zhou J, Zou D, et al. Treatment of relapsed or refractory classical hodgkin lymphoma with the anti-PD-1, Tislelizumab: results of a phase 2, single-arm, multicenter study. Leukemia. 2020;34:533–42. 31520078 10.1038/s41375-019-0545-2 PMC7214259

[56] Shumilov E, Wurm-Kuczera R, Kerkhoff A, Wang M, Melchardt T, Holtick U et al. Safety and Efficacy of Glofitamab for Relapsed/Refractory Large B-Cell Lymphoma in a Multinational Real-World Study. Blood Adv. 2024;bloodadvances.2024014903.10.1182/bloodadvances.202401490339661985

[57] Dahl JR, Weier A, Winter C, Hintze M, Rothhammer V, Tsaktanis T, et al. Modulator of VRAC current 1 is a potential target antigen in multiple sclerosis. Neurol Neuroimmunol Neuroinflamm. 2025;12:e200374. 39933126 10.1212/NXI.0000000000200374 PMC11839221

[58] Brudno JN, Maric I, Hartman SD, Rose JJ, Wang M, Lam N, et al. T cells genetically modified to express an Anti-B-Cell maturation antigen chimeric antigen receptor cause remissions of Poor-Prognosis relapsed multiple myeloma. J Clin Oncol. 2018;36:2267–80. 29812997 10.1200/JCO.2018.77.8084 PMC6067798

[59] Goodrich A. Advanced practice perspectives on preventing and managing tumor Lysis syndrome and neutropenia in chronic lymphocytic leukemia. J Adv Pract Oncol. 2021;12:59–70. 33552662 10.6004/jadpro.2021.12.1.5 PMC7844191

[60] Lycke J, Svenningsson A. Long-term treatment with anti-CD20 monoclonal antibodies is untenable because of risk: commentary. Mult Scler. 2022;28:1177–8. 35678609 10.1177/13524585221101138 PMC9189590

[61] Ram R, Ben-Bassat I, Shpilberg O, Polliack A, Raanani P. The late adverse events of rituximab therapy–rare but there! Leuk Lymphoma. 2009;50:1083–95. 19399690 10.1080/10428190902934944

[62] Yu W, Li P, Zhou L, Yang M, Ye S, Zhu D et al. A phase 1 trial of prizloncabtagene autoleucel, a CD19/CD20 CAR T-cell therapy for relapsed/refractory B-cell non-Hodgkin lymphoma. Blood. 2025;blood.2024026401. 10.1182/blood.2024026401 39813680

[63] Slamon DJ, Leyland-Jones B, Shak S, Fuchs H, Paton V, Bajamonde A, et al. Use of chemotherapy plus a monoclonal antibody against HER2 for metastatic breast cancer that overexpresses HER2. N Engl J Med. 2001;344:783–92. 11248153 10.1056/NEJM200103153441101

[64] Wiedmeier-Nutor J, Leis J. Chronic lymphocytic leukemia: chemotherapy free and other novel therapies including CAR T. Curr Treat Options Oncol. 2022;23:904–19. 35435617 10.1007/s11864-022-00953-5

[65] Zhao Z, Grégoire C, Oliveira B, Chung K, Melenhorst JJ. Challenges and opportunities of CAR T-cell therapies for CLL. Semin Hematol. 2023;60:25–33. 37080707 10.1053/j.seminhematol.2023.01.002

[66] Abbasi S, Totmaj MA, Abbasi M, Hajazimian S, Goleij P, Behroozi J, et al. Chimeric antigen receptor T (CAR-T) cells: novel cell therapy for hematological malignancies. Cancer Med. 2023;12:7844–58. 36583504 10.1002/cam4.5551 PMC10134288

[67] Fischer JW, Bhattarai NCAR-T, Cell Therapy. Mechanism, management, and mitigation of inflammatory toxicities. Front Immunol. 2021;12:693016. 34220853 10.3389/fimmu.2021.693016 PMC8250150

[68] Mancikova V, Peschelova H, Kozlova V, Ledererova A, Ladungova A, Verner J, et al. Performance of anti-CD19 chimeric antigen receptor T cells in genetically defined classes of chronic lymphocytic leukemia. J Immunother Cancer. 2020;8:e000471. 32217767 10.1136/jitc-2019-000471 PMC7206910

[69] Yin Z, Zhang Y, Wang X. Advances in chimeric antigen receptor T-cell therapy for B-cell non-Hodgkin lymphoma. Biomark Res. 2021;9:58. 34256851 10.1186/s40364-021-00309-5 PMC8278776

[70] Kochenderfer JN, Dudley ME, Kassim SH, Somerville RPT, Carpenter RO, Stetler-Stevenson M, et al. Chemotherapy-refractory diffuse large B-cell lymphoma and indolent B-cell malignancies can be effectively treated with autologous T cells expressing an anti-CD19 chimeric antigen receptor. J Clin Oncol. 2015;33:540–9. 25154820 10.1200/JCO.2014.56.2025 PMC4322257

[71] Carpenito C, Milone MC, Hassan R, Simonet JC, Lakhal M, Suhoski MM, et al. Control of large, established tumor xenografts with genetically retargeted human T cells containing CD28 and CD137 domains. Proc Natl Acad Sci U S A. 2009;106:3360–5. 19211796 10.1073/pnas.0813101106 PMC2651342

[72] Kittai AS, Huang Y, Miller S, Allan JN, Bhat SA, Bond DA, et al. Outcomes of patients with Richter transformation who received no prior chemoimmunotherapy for their CLL. Blood Cancer J. 2025;15:23. 39979241 10.1038/s41408-025-01236-6 PMC11842760

[73] Bock TJ, Colonne CK, Fiorenza S, Turtle CJ. Outcome correlates of approved CD19-targeted CAR T cells for large B cell lymphoma. Nat Rev Clin Oncol. 2025. 10.1038/s41571-025-00992-5 39966627

[74] Todorovic Z, Todorovic D, Markovic V, Ladjevac N, Zdravkovic N, Djurdjevic P, et al. CAR T cell therapy for chronic lymphocytic leukemia: successes and shortcomings. Curr Oncol. 2022;29:3647–57. 35621683 10.3390/curroncol29050293 PMC9139644

[75] Agarwal S, Aznar MA, Rech AJ, Good CR, Kuramitsu S, Da T, et al. Deletion of the inhibitory co-receptor CTLA-4 enhances and invigorates chimeric antigen receptor T cells. Immunity. 2023;56:2388–e24079. 37776850 10.1016/j.immuni.2023.09.001 PMC10591801

[76] Chandrasekaran S, Funk CR, Kleber T, Paulos CM, Shanmugam M, Waller EK. Strategies to overcome failures in T-Cell immunotherapies by targeting PI3K-δ and -γ. Front Immunol. 2021;12:718621. 34512641 10.3389/fimmu.2021.718621 PMC8427697

[77] Funk CR, Wang S, Chen KZ, Waller A, Sharma A, Edgar CL, et al. PI3Kδ/γ Inhibition promotes human CART cell epigenetic and metabolic reprogramming to enhance antitumor cytotoxicity. Blood. 2022;139:523–37. 35084470 10.1182/blood.2021011597 PMC8796652

[78] Grosser R, Cherkassky L, Chintala N, Adusumilli PS. Combination immunotherapy with CAR T cells and checkpoint Blockade for the treatment of solid tumors. Cancer Cell. 2019;36:471–82. 31715131 10.1016/j.ccell.2019.09.006 PMC7171534

[79] Locke FL, Ghobadi A, Jacobson CA, Miklos DB, Lekakis LJ, Oluwole OO, et al. Long-term safety and activity of Axicabtagene Ciloleucel in refractory large B-cell lymphoma (ZUMA-1): a single-arm, multicentre, phase 1–2 trial. Lancet Oncol. 2019;20:31–42. 30518502 10.1016/S1470-2045(18)30864-7 PMC6733402

[80] Fraietta JA, Beckwith KA, Patel PR, Ruella M, Zheng Z, Barrett DM, et al. Ibrutinib enhances chimeric antigen receptor T-cell engraftment and efficacy in leukemia. Blood. 2016;127:1117–27. 26813675 10.1182/blood-2015-11-679134 PMC4778162

[81] Gill S, Vides V, Frey NV, Hexner EO, Metzger S, O’Brien M, et al. Anti-CD19 CAR T cells in combination with ibrutinib for the treatment of chronic lymphocytic leukemia. Blood Adv. 2022;6:5774–85. 35349631 10.1182/bloodadvances.2022007317 PMC9647791

[82] Misawa K, Bhat H, Adusumilli PS, Hou Z. Combinational CAR T-cell therapy for solid tumors: requisites, rationales, and trials. Pharmacol Ther. 2025;266:108763. 39617146 10.1016/j.pharmthera.2024.108763 PMC11848936

[83] Uslu U, Castelli S, June CH. CAR T cell combination therapies to treat cancer. Cancer Cell. 2024;42:1319–25. 39059390 10.1016/j.ccell.2024.07.002

[84] Lee DW, Gardner R, Porter DL, Louis CU, Ahmed N, Jensen M, et al. Current concepts in the diagnosis and management of cytokine release syndrome. Blood. 2014;124:188–95. 24876563 10.1182/blood-2014-05-552729 PMC4093680

[85] Jain MD, Smith M, Shah NN. How I treat refractory CRS and ICANS after CAR T-cell therapy. Blood. 2023;141:2430–42. 36989488 10.1182/blood.2022017414 PMC10329191

[86] Neelapu SS, Tummala S, Kebriaei P, Wierda W, Gutierrez C, Locke FL, et al. Chimeric antigen receptor T-cell therapy - assessment and management of toxicities. Nat Rev Clin Oncol. 2018;15:47–62. 28925994 10.1038/nrclinonc.2017.148 PMC6733403

[87] Yang C, You J, Wang Y, Chen S, Tang Y, Chen H, et al. TLS and immune cell profiling: Immunomodulatory effects of immunochemotherapy on tumor microenvironment in resectable stage III NSCLC. Front Immunol. 2024;15:1499731. 39726591 10.3389/fimmu.2024.1499731 PMC11670196

[88] Nagle SJ, Murphree C, Raess PW, Schachter L, Chen A, Hayes-Lattin B, et al. Prolonged hematologic toxicity following treatment with chimeric antigen receptor T cells in patients with hematologic malignancies. Am J Hematol. 2021;96:455–61. 33529419 10.1002/ajh.26113

[89] Gauthier J, Gazeau N, Hirayama AV, Hill JA, Wu V, Cearley A, et al. Impact of CD19 CAR T-cell product type on outcomes in relapsed or refractory aggressive B-NHL. Blood. 2022;139:3722–31. 35439295 10.1182/blood.2021014497 PMC9247364

[90] Wudhikarn K, Palomba ML, Pennisi M, Garcia-Recio M, Flynn JR, Devlin SM, et al. Infection during the first year in patients treated with CD19 CAR T cells for diffuse large B cell lymphoma. Blood Cancer J. 2020;10:79. 32759935 10.1038/s41408-020-00346-7 PMC7405315

[91] Logue JM, Zucchetti E, Bachmeier CA, Krivenko GS, Larson V, Ninh D, et al. Immune reconstitution and associated infections following Axicabtagene Ciloleucel in relapsed or refractory large B-cell lymphoma. Haematologica. 2021;106:978–86. 32327504 10.3324/haematol.2019.238634 PMC8017820

[92] Braun LM, Giesler S, Andrieux G, Riemer R, Talvard-Balland N, Duquesne S, et al. Adiponectin reduces immune checkpoint inhibitor-induced inflammation without blocking anti-tumor immunity. Cancer Cell. 2025;43:269–e29119. 39933899 10.1016/j.ccell.2025.01.004

[93] Yan T, Long M, Liu C, Zhang J, Wei X, Li F, et al. Immune-related adverse events with PD-1/PD-L1 inhibitors: insights from a real-world cohort of 2523 patients. Front Pharmacol. 2025;16:1519082. 39959424 10.3389/fphar.2025.1519082 PMC11825824

[94] Othus M, Patel SP, Chae YK, Dietrich E, Streicher H, Sharon E et al. First cycle toxicity and survival in patients with rare cancers treated with checkpoint inhibitors. J Natl Cancer Inst. 2024;djae297. 10.1093/jnci/djae297 PMC11972677 39565908

[95] Gockeln L, Wirsdörfer F, Jendrossek V. CD73/adenosine dynamics in treatment-induced pneumonitis: balancing efficacy with risks of adverse events in combined radio-immunotherapies. Front Cell Dev Biol. 2024;12:1471072. 39872847 10.3389/fcell.2024.1471072 PMC11769960

[96] Gamero MT, Patel A, Storozynsky E. The good (Tumor Killing) and the bad (Cardiovascular Complications) of Immunologic checkpoint inhibitors. Curr Cardiol Rep. 2024;26:1487–98. 39441327 10.1007/s11886-024-02147-x PMC11668830

[97] Xu Q, Li X, Yuan Y, Liang G, Hu Z, Zhang W, et al. Development and validation of a nomogram for predicting immune-mediated colitis in lung cancer patients treated with immune checkpoint inhibitors: a retrospective cohort study in China. Front Immunol. 2025;16:1510053. 39949779 10.3389/fimmu.2025.1510053 PMC11821966

[98] Asnani M, Hayer KE, Naqvi AS, Zheng S, Yang SY, Oldridge D, et al. Retention of CD19 intron 2 contributes to CART-19 resistance in leukemias with subclonal frameshift mutations in CD19. Leukemia. 2020;34:1202–7. 31591467 10.1038/s41375-019-0580-z PMC7214268

[99] Tumuluru S, Godfrey JK, Cooper A, Yu J, Chen X, MacNabb B et al. Integrative genomic analysis of DLBCL identifies immune environments associated with bispecific antibody response. Blood. 2025;blood.2024025355. 10.1182/blood.2024025355 PMC12163739 39869833

[100] Choe JH, Watchmaker PB, Simic MS, Gilbert RD, Li AW, Krasnow NA, et al. SynNotch-CAR T cells overcome challenges of specificity, heterogeneity, and persistence in treating glioblastoma. Sci Transl Med. 2021;13:eabe7378. 33910979 10.1126/scitranslmed.abe7378 PMC8362330

[101] Prinzing B, Zebley CC, Petersen CT, Fan Y, Anido AA, Yi Z, et al. Deleting DNMT3A in CAR T cells prevents exhaustion and enhances antitumor activity. Sci Transl Med. 2021;13:eabh0272. 34788079 10.1126/scitranslmed.abh0272 PMC8733956

[102] Coorens THH, Collord G, Treger TD, Adams S, Mitchell E, Newman B, et al. Clonal origin of KMT2A wild-type lineage-switch leukemia following CAR-T cell and blinatumomab therapy. Nat Cancer. 2023;4:1095–101. 37474833 10.1038/s43018-023-00604-0 PMC10447231

[103] Mei H, Li C, Jiang H, Zhao X, Huang Z, Jin D, et al. A bispecific CAR-T cell therapy targeting BCMA and CD38 in relapsed or refractory multiple myeloma. J Hematol Oncol. 2021;14:161. 34627333 10.1186/s13045-021-01170-7 PMC8501733

[104] Spiegel JY, Patel S, Muffly L, Hossain NM, Oak J, Baird JH, et al. CAR T cells with dual targeting of CD19 and CD22 in adult patients with recurrent or refractory B cell malignancies: a phase 1 trial. Nat Med. 2021;27:1419–31. 34312556 10.1038/s41591-021-01436-0 PMC8363505

[105] Tang L, Pan S, Wei X, Xu X, Wei Q. Arming CAR-T cells with cytokines and more: innovations in the fourth-generation CAR-T development. Mol Ther. 2023;31:3146–62. 37803832 10.1016/j.ymthe.2023.09.021 PMC10638038

[106] Albelda SM. CAR T cell therapy for patients with solid tumours: key lessons to learn and unlearn. Nat Rev Clin Oncol. 2024;21:47–66. 37904019 10.1038/s41571-023-00832-4

[107] Yang Y, Peng H, Wang J, Li F. New insights into CAR T-cell hematological toxicities: manifestations, mechanisms, and effective management strategies. Exp Hematol Oncol. 2024;13:110. 39521987 10.1186/s40164-024-00573-9 PMC11549815

[108] Sharma R, Suravarjhula L, Banerjee M, Kumar G, Kumar N. Chimeric antigen receptor T-cell therapy in cancer: A critical review. Curr Drug Res Rev. 2023;15:241–61. 36825696 10.2174/2589977515666230220092125

[109] Pan K, Farrukh H, Chittepu VCSR, Xu H, Pan C-X, Zhu Z. CAR race to cancer immunotherapy: from CAR T, CAR NK to CAR macrophage therapy. J Exp Clin Cancer Res. 2022;41:119. 35361234 10.1186/s13046-022-02327-z PMC8969382

[110] Xie G, Dong H, Liang Y, Ham JD, Rizwan R, Chen J. CAR-NK cells: A promising cellular immunotherapy for cancer. EBioMedicine. 2020;59:102975. 32853984 10.1016/j.ebiom.2020.102975 PMC7452675

[111] Gurney M, O’Reilly E, Corcoran S, Brophy S, Krawczyk J, Otto NM, et al. Concurrent transposon engineering and CRISPR/Cas9 genome editing of primary CLL-1 chimeric antigen receptor-natural killer cells. Cytotherapy. 2022;24:1087–94. 36050244 10.1016/j.jcyt.2022.07.008

[112] Gong Y, Klein Wolterink RGJ, Wang J, Bos GMJ, Germeraad WTV. Chimeric antigen receptor natural killer (CAR-NK) cell design and engineering for cancer therapy. J Hematol Oncol. 2021;14:73. 33933160 10.1186/s13045-021-01083-5 PMC8088725

[113] Dagher OK, Posey AD. Forks in the road for CAR T and CAR NK cell cancer therapies. Nat Immunol. 2023;24:1994–2007. 38012406 10.1038/s41590-023-01659-y PMC12798859

[114] June CH, O’Connor RS, Kawalekar OU, Ghassemi S, Milone MC. CAR T cell immunotherapy for human cancer. Science. 2018;359:1361–5. 29567707 10.1126/science.aar6711

[115] Trapani JA. Granzymes: a family of lymphocyte granule Serine proteases. Genome Biol. 2001;2:REVIEWS3014. 11790262 10.1186/gb-2001-2-12-reviews3014 PMC138995

[116] Kerbauy LN, Marin ND, Kaplan M, Banerjee PP, Berrien-Elliott MM, Becker-Hapak M, et al. Combining AFM13, a bispecific CD30/CD16 antibody, with Cytokine-Activated blood and cord blood-Derived NK cells facilitates CAR-like responses against CD30 + Malignancies. Clin Cancer Res. 2021;27:3744–56. 33986022 10.1158/1078-0432.CCR-21-0164 PMC8254785

[117] Zhang L, Meng Y, Feng X, Han Z. CAR-NK cells for cancer immunotherapy: from bench to bedside. Biomark Res. 2022;10:12. 35303962 10.1186/s40364-022-00364-6 PMC8932134

[118] Herrera L, Santos S, Vesga MA, Carrascosa T, Garcia-Ruiz JC, Pérez-Martínez A, et al. The race of CAR therapies: CAR-NK cells for fighting B-Cell hematological cancers. Cancers (Basel). 2021;13:5418. 34771581 10.3390/cancers13215418 PMC8582420

[119] Shi Y, Hao D, Qian H, Tao Z. Natural killer cell-based cancer immunotherapy: from basics to clinical trials. Exp Hematol Oncol. 2024;13:101. 39415291 10.1186/s40164-024-00561-z PMC11484118

[120] Li Y, Yin J, Li T, Huang S, Yan H, Leavenworth J, et al. NK cell-based cancer immunotherapy: from basic biology to clinical application. Sci China Life Sci. 2015;58:1233–45. 26588912 10.1007/s11427-015-4970-9

[121] Zhong Y, Liu J. Emerging roles of CAR-NK cell therapies in tumor immunotherapy: current status and future directions. Cell Death Discov. 2024;10:318. 38987565 10.1038/s41420-024-02077-1 PMC11236993

[122] Marin D, Li Y, Basar R, Rafei H, Daher M, Dou J, et al. Safety, efficacy and determinants of response of allogeneic CD19-specific CAR-NK cells in CD19 + B cell tumors: a phase 1/2 trial. Nat Med. 2024;30:772–84. 38238616 10.1038/s41591-023-02785-8 PMC10957466

[123] Liu E, Marin D, Banerjee P, et al. Use of CAR-Transduced Natural Killer Cells in CD19-Positive Lymphoid Tumors. N Engl J Med. 2020;382(6):545-553. 10.1056/NEJMoa1910607 PMC7101242 32023374

[124] Marofi F, Rahman HS, Thangavelu L, Dorofeev A, Bayas-Morejón F, Shirafkan N, et al. Renaissance of armored immune effector cells, CAR-NK cells, brings the higher hope for successful cancer therapy. Stem Cell Res Ther. 2021;12:200. 33752707 10.1186/s13287-021-02251-7 PMC7983395

[125] Rafei H, Daher M, Rezvani K. Chimeric antigen receptor (CAR) natural killer (NK)-cell therapy: leveraging the power of innate immunity. Br J Haematol. 2021;193:216–30. 33216984 10.1111/bjh.17186 PMC9942693

[126] Maalej KM, Merhi M, Inchakalody VP, Mestiri S, Alam M, Maccalli C, et al. CAR-cell therapy in the era of solid tumor treatment: current challenges and emerging therapeutic advances. Mol Cancer. 2023;22:20. 36717905 10.1186/s12943-023-01723-z PMC9885707

[127] Klichinsky M, Ruella M, Shestova O, Lu XM, Best A, Zeeman M, et al. Human chimeric antigen receptor macrophages for cancer immunotherapy. Nat Biotechnol. 2020;38:947–53. 32361713 10.1038/s41587-020-0462-y PMC7883632

[128] Sloas C, Gill S, Klichinsky M. Engineered CAR-Macrophages as adoptive immunotherapies for solid tumors. Front Immunol. 2021;12:783305. 34899748 10.3389/fimmu.2021.783305 PMC8652144

[129] Lei A, Yu H, Lu S, Lu H, Ding X, Tan T, et al. A second-generation M1-polarized CAR macrophage with antitumor efficacy. Nat Immunol. 2024;25:102–16. 38012418 10.1038/s41590-023-01687-8

[130] Santoni M, Massari F, Santoni G, Cimadamore A, Montironi R, Battelli N. Re: human chimeric antigen receptor macrophages for cancer immunotherapy. Eur Urol. 2021;79:887–9. 33549360 10.1016/j.eururo.2021.01.025

[131] Lu J, Ma Y, Li Q, Xu Y, Xue Y, Xu S. CAR macrophages: a promising novel immunotherapy for solid tumors and beyond. Biomark Res. 2024;12:86. 39175095 10.1186/s40364-024-00637-2 PMC11342599

[132] Chen Y, Yu Z, Tan X, Jiang H, Xu Z, Fang Y, et al. CAR-macrophage: A new immunotherapy candidate against solid tumors. Biomed Pharmacother. 2021;139:111605. 33901872 10.1016/j.biopha.2021.111605

[133] Falchi L, Vardhana SA, Salles GA. Bispecific antibodies for the treatment of B-cell lymphoma: promises, unknowns, and opportunities. Blood. 2023;141:467–80. 36322929 10.1182/blood.2021011994 PMC9936308

[134] Tian Z, Liu M, Zhang Y, Wang X. Bispecific T cell engagers: an emerging therapy for management of hematologic malignancies. J Hematol Oncol. 2021;14:75. 33941237 10.1186/s13045-021-01084-4 PMC8091790

[135] Moon D, Tae N, Park Y, Lee S-W, Kim DH. Development of bispecific antibody for cancer immunotherapy: focus on T cell engaging antibody. Immune Netw. 2022;22:e4. 35291652 10.4110/in.2022.22.e4 PMC8901699

[136] Labrijn AF, Janmaat ML, Reichert JM, Parren PWHI. Bispecific antibodies: a mechanistic review of the pipeline. Nat Rev Drug Discov. 2019;18:585–608. 31175342 10.1038/s41573-019-0028-1

[137] Wong R, Pepper C, Brennan P, Nagorsen D, Man S, Fegan C. Blinatumomab induces autologous T-cell killing of chronic lymphocytic leukemia cells. Haematologica. 2013;98:1930–8. 23812940 10.3324/haematol.2012.082248 PMC3857012

[138] McWilliams EM, Mele JM, Cheney C, Timmerman EA, Fiazuddin F, Strattan EJ, et al. Therapeutic CD94/NKG2A Blockade improves natural killer cell dysfunction in chronic lymphocytic leukemia. Oncoimmunology. 2016;5:e1226720. 27853650 10.1080/2162402X.2016.1226720 PMC5087289

[139] Vyas M, Schneider A-C, Shatnyeva O, Reiners KS, Tawadros S, Kloess S, et al. Mono- and dual-targeting triplebodies activate natural killer cells and have anti-tumor activity in vitro and in vivo against chronic lymphocytic leukemia. Oncoimmunology. 2016;5:e1211220. 27757305 10.1080/2162402X.2016.1211220 PMC5049355

[140] Byrd JC, Pagel JM, Awan FT, Forero A, Flinn IW, Deauna-Limayo DP, et al. A phase 1 study evaluating the safety and tolerability of Otlertuzumab, an anti-CD37 mono-specific ADAPTIR therapeutic protein in chronic lymphocytic leukemia. Blood. 2014;123:1302–8. 24381226 10.1182/blood-2013-07-512137 PMC3938145

[141] Klupsch K, Baeriswyl V, Scholz R, Dannenberg J, Santimaria R, Senn D, et al. COVA4231, a potent CD3/CD33 bispecific fynomab with IgG-like pharmacokinetics for the treatment of acute myeloid leukemia. Leukemia. 2019;33:805–8. 30206306 10.1038/s41375-018-0249-z

[142] Choi MY, Widhopf GF, Ghia EM, Kidwell RL, Hasan MK, Yu J, et al. Phase I trial: Cirmtuzumab inhibits ROR1 signaling and stemness signatures in patients with chronic lymphocytic leukemia. Cell Stem Cell. 2018;22:951–e9593. 29859176 10.1016/j.stem.2018.05.018 PMC7001723

[143] Liu X, Zhao J, Guo X, Song Y. CD20 × CD3 bispecific antibodies for lymphoma therapy: latest updates from ASCO 2023 annual meeting. J Hematol Oncol. 2023;16:90. 37537626 10.1186/s13045-023-01488-4 PMC10401875

[144] Linton KM, Vitolo U, Jurczak W, Lugtenburg PJ, Gyan E, Sureda A, et al. Epcoritamab monotherapy in patients with relapsed or refractory follicular lymphoma (EPCORE NHL-1): a phase 2 cohort of a single-arm, multicentre study. Lancet Haematol. 2024;11:e593–605. 38889737 10.1016/S2352-3026(24)00166-2

[145] Sun LL, Ellerman D, Mathieu M, Hristopoulos M, Chen X, Li Y, et al. Anti-CD20/CD3 T cell-dependent bispecific antibody for the treatment of B cell malignancies. Sci Transl Med. 2015;7:287ra70. 25972002 10.1126/scitranslmed.aaa4802

[146] Lu C-Y, Chen GJ, Tai P-H, Yang Y-C, Hsu Y-S, Chang M, et al. Tetravalent anti-CD20/CD3 bispecific antibody for the treatment of B cell lymphoma. Biochem Biophys Res Commun. 2016;473:808–13. 27040766 10.1016/j.bbrc.2016.03.124

[147] Li Y, Li C, Lv K, Wang S, Li F. Efficacy and safety of ibrutinib as monotherapy or combination therapy in relapsed/refractory diffuse large B-cell lymphoma (R/R DLBCL): A systematic review and Meta-analysis. Am J Ther. 2025;32:e5–16. 39413356 10.1097/MJT.0000000000001831 PMC11698132

[148] Thieblemont C, Karimi YH, Ghesquieres H, Cheah CY, Clausen MR, Cunningham D, et al. Epcoritamab in relapsed/refractory large B-cell lymphoma: 2-year follow-up from the pivotal EPCORE NHL-1 trial. Leukemia. 2024;38:2653–62. 39322711 10.1038/s41375-024-02410-8 PMC11588654

[149] Topp MS, Gökbuget N, Stein AS, Zugmaier G, O’Brien S, Bargou RC, et al. Safety and activity of blinatumomab for adult patients with relapsed or refractory B-precursor acute lymphoblastic leukaemia: a multicentre, single-arm, phase 2 study. Lancet Oncol. 2015;16:57–66. 25524800 10.1016/S1470-2045(14)71170-2

[150] Bangolo A, Amoozgar B, Mansour C, Zhang L, Gill S, Ip A, et al. Comprehensive review of early and late toxicities in CAR T-Cell therapy and bispecific antibody treatments for hematologic malignancies. Cancers (Basel). 2025;17:282. 39858064 10.3390/cancers17020282 PMC11764151

[151] Goebeler M-E, Stuhler G, Bargou R. Bispecific and multispecific antibodies in oncology: opportunities and challenges. Nat Rev Clin Oncol. 2024;21:539–60. 38822215 10.1038/s41571-024-00905-y

[152] Kontermann RE, Brinkmann U. Bispecific antibodies. Drug Discov Today. 2015;20:838–47. 25728220 10.1016/j.drudis.2015.02.008

[153] Long M, Mims AS, Li Z. Factors affecting the cancer immunotherapeutic efficacy of T cell bispecific antibodies and strategies for improvement. Immunol Invest. 2022;51:2176–214. 36259611 10.1080/08820139.2022.2131569

[154] Kontermann RE. Dual targeting strategies with bispecific antibodies. MAbs. 2012;4:182–97. 22453100 10.4161/mabs.4.2.19000 PMC3361654

[155] Brinkmann U, Kontermann RE. Bispecific antibodies. Science. 2021;372:916–7. 34045345 10.1126/science.abg1209

[156] Yao Y, Hu Y, Wang F. Trispecific antibodies for cancer immunotherapy. Immunology. 2023;169:389–99. 36855956 10.1111/imm.13636

[157] Zhao L, Li S, Wei X, Qi X, Liu D, Liu L, et al. A novel CD19/CD22/CD3 trispecific antibody enhances therapeutic efficacy and overcomes immune escape against B-ALL. Blood. 2022;140:1790–802. 35981465 10.1182/blood.2022016243

[158] Müller D. Optimized CD19/CD22/CD3 antibody. Blood. 2022;140:1750–1. 36264591 10.1182/blood.2022018081

[159] Thomas A, Teicher BA, Hassan R. Antibody-drug conjugates for cancer therapy. Lancet Oncol. 2016;17:e254–62. 27299281 10.1016/S1470-2045(16)30030-4 PMC6601617

[160] Jin Y, Schladetsch MA, Huang X, Balunas MJ, Wiemer AJ. Stepping forward in antibody-drug conjugate development. Pharmacol Ther. 2022;229:107917. 34171334 10.1016/j.pharmthera.2021.107917 PMC8702582

[161] Birrer MJ, Moore KN, Betella I, Bates RC. Antibody-Drug Conjugate-Based therapeutics: state of the science. J Natl Cancer Inst. 2019;111:538–49. 30859213 10.1093/jnci/djz035

[162] Tsuchikama K, An Z. Antibody-drug conjugates: recent advances in conjugation and linker chemistries. Protein Cell. 2018;9:33–46. 27743348 10.1007/s13238-016-0323-0 PMC5777969

[163] Fu Z, Li S, Han S, Shi C, Zhang Y. Antibody drug conjugate: the biological missile for targeted cancer therapy. Sig Transduct Target Ther. 2022;7:93. 10.1038/s41392-022-00947-7 PMC8941077 35318309

[164] Polson AG, Calemine-Fenaux J, Chan P, Chang W, Christensen E, Clark S, et al. Antibody-drug conjugates for the treatment of non-Hodgkin’s lymphoma: target and linker-drug selection. Cancer Res. 2009;69:2358–64. 19258515 10.1158/0008-5472.CAN-08-2250

[165] Harker-Murray P, Mauz-Körholz C, Leblanc T, Mascarin M, Michel G, Cooper S, et al. Nivolumab and Brentuximab Vedotin with or without Bendamustine for R/R hodgkin lymphoma in children, adolescents, and young adults. Blood. 2023;141:2075–84. 36564047 10.1182/blood.2022017118 PMC10646780

[166] Fornecker L-M, Lazarovici J, Aurer I, Casasnovas R-O, Gac A-C, Bonnet C, et al. Brentuximab Vedotin plus AVD for First-Line treatment of Early-Stage unfavorable hodgkin lymphoma (BREACH): A multicenter, Open-Label, randomized, phase II trial. J Clin Oncol. 2023;41:327–35. 35867960 10.1200/JCO.21.01281

[167] Nakashima M, Uchimaru K. CD30 expression and its functions during the disease progression of adult T-Cell leukemia/lymphoma. Int J Mol Sci. 2023;24:8731. 37240076 10.3390/ijms24108731 PMC10218159

[168] Moskowitz CH, Walewski J, Nademanee A, Masszi T, Agura E, Holowiecki J, et al. Five-year PFS from the AETHERA trial of Brentuximab Vedotin for hodgkin lymphoma at high risk of progression or relapse. Blood. 2018;132:2639–42. 30266774 10.1182/blood-2018-07-861641

[169] Tilly H, Morschhauser F, Sehn LH, Friedberg JW, Trněný M, Sharman JP, et al. Polatuzumab Vedotin in previously untreated diffuse large B-Cell lymphoma. N Engl J Med. 2022;386:351–63. 34904799 10.1056/NEJMoa2115304 PMC11702892

[170] Sehn LH, Herrera AF, Flowers CR, Kamdar MK, McMillan A, Hertzberg M, et al. Polatuzumab Vedotin in relapsed or refractory diffuse large B-Cell lymphoma. J Clin Oncol. 2020;38:155–65. 31693429 10.1200/JCO.19.00172 PMC7032881

[171] Xu B. Loncastuximab Tesirine: an effective therapy for relapsed or refractory diffuse large B-cell lymphoma. Eur J Clin Pharmacol. 2022;78:707–19. 35061047 10.1007/s00228-021-03253-3

[172] Caimi PF, Ai W, Alderuccio JP, Ardeshna KM, Hamadani M, Hess B, et al. Loncastuximab Tesirine in relapsed or refractory diffuse large B-cell lymphoma (LOTIS-2): a multicentre, open-label, single-arm, phase 2 trial. Lancet Oncol. 2021;22:790–800. 33989558 10.1016/S1470-2045(21)00139-X

[173] Juárez-Salcedo LM, Nimkar S, Corazón AM, Dalia S. Loncastuximab Tesirine in the treatment of relapsed or refractory diffuse large B-Cell lymphoma. Int J Mol Sci. 2024;25:7580. 39062823 10.3390/ijms25147580 PMC11276998

[174] Caimi PF, Ai WZ, Alderuccio JP, Ardeshna KM, Hamadani M, Hess B, et al. Loncastuximab Tesirine in relapsed/refractory diffuse large B-cell lymphoma: long-term efficacy and safety from the phase II LOTIS-2 study. Haematologica. 2024;109:1184–93. 37646659 10.3324/haematol.2023.283459 PMC10985439

[175] Lambert J, Pautas C, Terré C, Raffoux E, Turlure P, Caillot D, et al. Gemtuzumab Ozogamicin for de Novo acute myeloid leukemia: final efficacy and safety updates from the open-label, phase III ALFA-0701 trial. Haematologica. 2019;104:113–9. 30076173 10.3324/haematol.2018.188888 PMC6312010

[176] Hills RK, Castaigne S, Appelbaum FR, Delaunay J, Petersdorf S, Othus M, et al. Addition of Gemtuzumab Ozogamicin to induction chemotherapy in adult patients with acute myeloid leukaemia: a meta-analysis of individual patient data from randomised controlled trials. Lancet Oncol. 2014;15:986–96. 25008258 10.1016/S1470-2045(14)70281-5 PMC4137593

[177] Castaigne S, Pautas C, Terré C, Raffoux E, Bordessoule D, Bastie J-N, et al. Effect of Gemtuzumab Ozogamicin on survival of adult patients with de-novo acute myeloid leukaemia (ALFA-0701): a randomised, open-label, phase 3 study. Lancet. 2012;379:1508–16. 22482940 10.1016/S0140-6736(12)60485-1

[178] Kantarjian HM, DeAngelo DJ, Stelljes M, Martinelli G, Liedtke M, Stock W, et al. Inotuzumab Ozogamicin versus standard therapy for acute lymphoblastic leukemia. N Engl J Med. 2016;375:740–53. 27292104 10.1056/NEJMoa1509277 PMC5594743

[179] Kantarjian HM, DeAngelo DJ, Stelljes M, Liedtke M, Stock W, Gökbuget N, et al. Inotuzumab Ozogamicin versus standard of care in relapsed or refractory acute lymphoblastic leukemia: final report and long-term survival follow-up from the randomized, phase 3 INO-VATE study. Cancer. 2019;125:2474–87. 30920645 10.1002/cncr.32116 PMC6618133

[180] Dhillon S. Moxetumomab pasudotox: first global approval. Drugs. 2018;78:1763–7. 30357593 10.1007/s40265-018-1000-9 PMC6323103

[181] Lin AY, Dinner SN. Moxetumomab pasudotox for hairy cell leukemia: preclinical development to FDA approval. Blood Adv. 2019;3:2905–10. 31594764 10.1182/bloodadvances.2019000507 PMC6784515

[182] Markham A. Belantamab Mafodotin: first approval. Drugs. 2020;80:1607–13. 32936437 10.1007/s40265-020-01404-x

[183] Hungria V, Mateos M-V. Belantamab Mafodotin, bortezomib, and dexamethasone for multiple myeloma. Reply. N Engl J Med. 2024;391:1364–5. 39383466 10.1056/NEJMc2410841

[184] Wang J, Zhang X, Zhou Z, Liu Y, Yu L, Jia L, et al. A novel adoptive synthetic TCR and antigen receptor (STAR) T-Cell therapy for B-Cell acute lymphoblastic leukemia. Am J Hematol. 2022;97:992–1004. 35491511 10.1002/ajh.26586

[185] Chen ZW. Comparative biology of gamma delta T cells. Sci Prog. 2002;85:347–58. 12661423 10.3184/003685002783238762 PMC10367533

[186] Chien YH, Jores R, Crowley MP. Recognition by gamma/delta T cells. Annu Rev Immunol. 1996;14:511–32. 8717523 10.1146/annurev.immunol.14.1.511

[187] Gentles AJ, Newman AM, Liu CL, Bratman SV, Feng W, Kim D, et al. The prognostic landscape of genes and infiltrating immune cells across human cancers. Nat Med. 2015;21:938–45. 26193342 10.1038/nm.3909 PMC4852857

[188] Silva-Santos B, Mensurado S, Coffelt SB. γδ T cells: pleiotropic immune effectors with therapeutic potential in cancer. Nat Rev Cancer. 2019;19:392–404. 31209264 10.1038/s41568-019-0153-5 PMC7614706

[189] Capsomidis A, Benthall G, Van Acker HH, Fisher J, Kramer AM, Abeln Z, et al. Chimeric antigen Receptor-Engineered human gamma delta T cells: enhanced cytotoxicity with retention of cross presentation. Mol Ther. 2018;26:354–65. 29310916 10.1016/j.ymthe.2017.12.001 PMC5835118

[190] Ma L, Feng Y, Zhou Z. A close look at current Γδ T-cell immunotherapy. Front Immunol. 2023;14:1140623. 37063836 10.3389/fimmu.2023.1140623 PMC10102511

[191] Ebina T. [Life-prolonging effect of Immunocell BAK (BRM activated killer) therapy–evidence based integrative medicine]. Gan Kagaku Ryoho. 2004;31:1643–5. 15553670

[192] Ebina T. [Non-small cell lung cancer–immunocell BAK (BRM activated killer) therapy]. Nihon Rinsho. 2006;64:1339–44. 16838654

[193] Ebina T, Fujimiya Y, Yamaguchi T, Ogama N, Sasaki H, Isono N, et al. The use of BRM-activated killer cells in adoptive immunotherapy: a pilot study with nine advanced cancer patients. Biotherapy. 1998;11:241–53. 9950100 10.1023/a:1008047628284

[194] Ebina T. [Local injection of BRM-activated killer cells into an abdominal wall tumor]. Gan Kagaku Ryoho. 2013;40:1507–9. 24231704

[195] Ebina T. [Hepatic intra-arterial infusion of biological response modifier (BRM)-activated killer immune lymphocytes to treat liver cancer]. Gan Kagaku Ryoho. 2012;39:1812–4. 23267895

[196] Ebina T, Ogama N, Shimanuki H, Kubota T, Isono N. Effector mechanism and clinical response of BAK (BRM-activated killer) immuno-cell therapy for maintaining satisfactory QOL of advanced cancer patients utilizing CD56-positive NIE (neuro-immune-endocrine) cells. Microbiol Immunol. 2001;45:403–11. 11471830 10.1111/j.1348-0421.2001.tb02638.x

[197] Urushizaki I. [Therapy by biological response modifiers]. Rinsho Ketsueki. 1989;30:1230–2. 2689681

